# Ex vivo-expanded human CD19^+^TIM-1^+^ regulatory B cells suppress immune responses in vivo and are dependent upon the TIM-1/STAT3 axis

**DOI:** 10.1038/s41467-022-30613-z

**Published:** 2022-06-03

**Authors:** S. Shankar, J. Stolp, S. C. Juvet, J. Beckett, P. S. Macklin, F. Issa, J. Hester, K. J. Wood

**Affiliations:** 1 Translational Research and Immunology Group, Nuffield Department of Surgical Sciences, University of Oxford, Oxford, UK; 2 Oxford Transplant Centre, Nuffield Department of Surgical Sciences, University of Oxford, Oxford, UK; 3 Nuffield Department of Medicine Research Building, Nuffield Department of Medicine, University of Oxford, Oxford, UK

**Keywords:** Tumour immunology, Immunosuppression, B cells, Allotransplantation

## Abstract

Regulatory B cells (Breg) are a heterogenous population with immune-modulating functions. The rarity of human IL-10^+^ Breg makes translational studies difficult. Here we report ex vivo expansion of human B cells with in vivo regulatory function (expBreg). CD154-stimulation of human CD19^+^ B cells drives >900-fold expansion of IL-10^+^ B cells that is maintained in culture for 14 days. Whilst expBreg-mediated suppressive function is partially dependent on IL-10 expression, CRISPR-mediated gene deletions demonstrate predominant roles for TIM-1 and CD154. TIM-1 regulates STAT3 signalling and modulates downstream suppressive function. In a clinically relevant humanised mouse model of skin transplantation, expBreg prolongs human allograft survival. Meanwhile, CD19^+^CD73^-^CD25^+^CD71^+^TIM-1^+^CD154^+^ Breg cells are enriched in the peripheral blood of human donors with cutaneous squamous cell carcinoma (SCC). TIM-1^+^ and pSTAT3^+^ B cells are also identified in B cell clusters within histological sections of human cutaneous SCC tumours. Our findings thus provide insights on Breg homoeostasis and present possible targets for Breg-related therapies.

## Abstract (web summary)

Human regulatory B (Breg) cells have been difficult to study due to their scarcity and heterogeneity. Here the authors expand human B cells to exert immunosuppressive function in vitro and in vivo, and to implicate the TIM-1/STAT3 axis for the regulation of their homoeostasis and function.

## Introduction

The importance of IL-10^+^ B regulatory cells (Breg) in both health and disease has rapidly become evident over the last decade^1–5^. Breg are able to control immune responses in animal models of transplantation and autoimmunity^6–10^, Breg populations are enriched in the peripheral blood of operationally tolerant human kidney transplant recipients^1,2^ and Breg markers have been recently reported to predict acute rejection and allograft dysfunction in transplantation^11^. Conversely, excessive regulation by Breg may contribute to the development of cancer and other diseases such as Human Immunodeficiency Virus (HIV-1)^12–15^.

Mouse Breg have been successfully induced and expanded to allow mechanistic interrogation and in vivo demonstration of suppressive function using adoptive transfer models of autoimmunity and transplant^16–18^. However, fundamental phenotypic and functional differences between mouse and human Breg, the rarity of human Breg in peripheral blood and difficulties with characterisation^3,4,19^ mean that in vivo investigation and the potential to target human Breg to augment or inhibit their function therapeutically remain elusive. Following isolation of IL-10^+^ Breg-enriched B-cell subsets from human tissue or blood, human Breg are often characterised by the property to express IL-10 when stimulated, as well as the ability to suppress human immune responses in vitro^3,4,20–22^. However, protocols to drive human Breg expansion to levels sufficient for application as a cellular therapy are lacking, as is functional evidence of their in vivo suppressive roles and stability. Our limited understanding of intracellular signalling pathways within endogenous human Breg subsets constrains the potential to therapeutically target human Breg in vivo.

Here we report ex vivo expansion of human IL-10^+^ B cells with in vivo regulatory function (expBreg). Gene knockouts demonstrate predominant roles for TIM-1 and CD154 in mediating suppressive function. Specifically, TIM-1 regulates STAT3 signalling, modulating downstream suppressive function. expBreg prolong human allograft survival in vivo and are associated with an increased percentage of human CD4^+^CD25^+^CD127^lo^ Treg within the allograft. expBreg express an endogenous human Breg phenotype, CD19^+^CD73^−^CD25^+^CD71^+^, and upregulate TIM-1 and CD154. Furthermore, CD19^+^TIM-1^+^ Breg are enriched in peripheral blood and tumours of human donors with cutaneous squamous cell carcinoma (SCC). Our findings provide strategies to develop CD19^+^TIM-1^+^ Breg cellular therapy and they identify potential therapeutic targets to modulate CD19^+^TIM-1^+^ Breg function across several disease states.

## Results

### CD154 stimulation drives ex vivo expansion of human IL-10^+^ B cells for up to 14 days

**Fig. 1:**
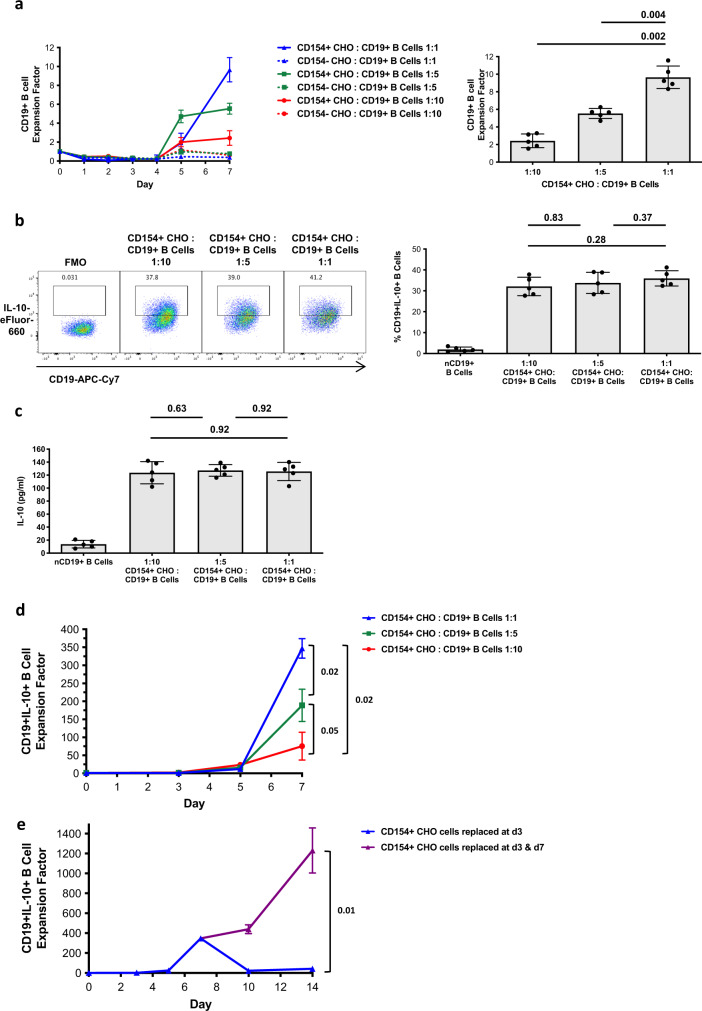
CD154 stimulation drives ex vivo expansion of human IL-10^+^ B cells for up to 14 days. **a** Human CD19^+^ B cells were co-cultured with CD154^+^ or CD154^−^ CHO cells for 7 days at different CHO:B-cell ratios and in the presence of IL-2, IL-4 and IL-10. Expansion factor is ratio of the number of live CD19^+^ B cells at each day to the original number of live CD19^+^ B cells on day 0 (expansion factor at day 0 = 1). Purity of CD19^+^ B cells >96% in all experiments. Data from experiments performed with cells from different healthy donors (*n* = 5) are presented. Each dot is an individual response. **b** Representative FACS plots and summarised data of IL-10 expression by human CD19^+^ B cells which were expanded at different CD154^+^ CHO cell:CD19^+^ B-cell ratios, are shown. Gating is on fluorescence-minus-one (FMO) controls. Data from experiments performed with cells from different healthy donors (*n* = 5) are presented. Each dot is an individual response. **c** Summarised data of IL-10 production by human CD19^+^ B cells, which were expanded at different CD154^+^ CHO cell-CD19^+^ B-cell ratios, are shown. Data from experiments performed with cells from different healthy donors (*n* = 5) are presented. Each dot is an individual response. **d** Increased proliferation and % increased IL-10 expression by expBreg following 7-day expansion results in >300-fold expansion of human CD19^+^IL-10^+^ B cells. Expansion factor is the ratio of the number of live CD19^+^IL-10^+^ B cells to the original number of live CD19^+^IL-10^+^ B cells on day 0 (expansion factor at day 0 = 1). Data from experiments performed with cells from different healthy donors (*n* = 3) are presented. **e** Human CD19^+^IL-10^+^ B-cell expansion could be maintained in culture for at least 14 days when CD154^+^ CHO cells were replaced at day-3 and day-7 within the ex vivo culture system, resulting in almost 900-fold expansion. Data from experiments performed with cells from different healthy donors (*n* = 3) are presented. Error bars in each panel represent Mean ± SD (**a**–**e**). One-way ANOVA with Tukey’s multiple comparisons test (**a**–**d**) and 2-tailed paired *t* test (**e**) were used. Source data are provided as a Source Data file.

Induction of IL-10 expression by human CD19^+^ B cells can be stimulated by CD154, a ligand which is upregulated by early-activated CD4^+^ T cells^20^. The CD154–CD40 interaction has been used to expand mouse splenic B10 cells in vitro^16^, and to induce IL-10 expression in rare human Breg populations which first have been isolated from peripheral blood^4,23^. To determine whether whole CD19^+^ B-cell populations present in peripheral human blood could be stimulated to generate large numbers of human IL-10^+^ B cells, we co-cultured human CD19^+^ B cells with irradiated CD154^+^ Chinese Hamster Ovary (CHO) cell line for 7 days ex vivo in the presence of cytokines IL-2, IL-4 and IL-10 (Fig. 1a). The rate of human CD19^+^ B-cell expansion correlated with the ratio of CD154^+^ CHO cells to CD19^+^ B cells, with greatest expansion at a ratio of 1:1 CD154^+^ CHO cells to CD19^+^ B cells (Fig. 1a). Expansion of human CD19^+^ B cells was also achieved when varying the cytokine combinations in co-cultures, but optimal rates were achieved with IL-2, IL-4 and IL-10 (Supplementary Fig. 1a).

6 h stimulation of expanded human CD19^+^ B cells with PMA & Ionomycin on day-7 of culture, demonstrated increased IL-10 expression and IL-10 production by CD19^+^ B cells following expansion (Fig. 1b, c). However, there was no difference in the percentage expression or production of IL-10 by expanded CD19^+^ B cells, when varying the ratio of CD154^+^ CHO cells to CD19^+^ B cells (Fig. 1b, c). Optimal expansion conditions consistently resulted in greater than 300-fold expansion of human CD19^+^IL-10^+^ B cells after 7 days in culture (Fig. 1d). Percentage cell death decreased by day-7 of co-culture (Supplementary Fig. 1b). Human CD19^+^IL-10^+^ B-cell expansion could be maintained in culture for at least 14 days when CD154^+^ CHO cells were regularly replaced within the ex vivo culture system, resulting in almost 900-fold expansion (Fig. 1e and Supplementary Fig. 1c). The frequency of human CD19^+^ B cells and IL-10 production decreased, and percentage cell death increased by day-14 if CD19^+^ B cells were not re-stimulated with CD154^+^ CHO cells at day-7 of co-culture, indicating that repeated CD154 stimulation was required to maintain human IL-10^+^ B-cell expansion (Supplementary Fig. 1d–f). Human CD19^+^IL-10^+^ B cells could therefore be generated, expanded and maintained in culture for at least 14 days.

### Expanded human CD19^+^IL-10^+^ B cells can suppress autologous CD4^+^ T-cell responses in vitro

**Fig. 2:**
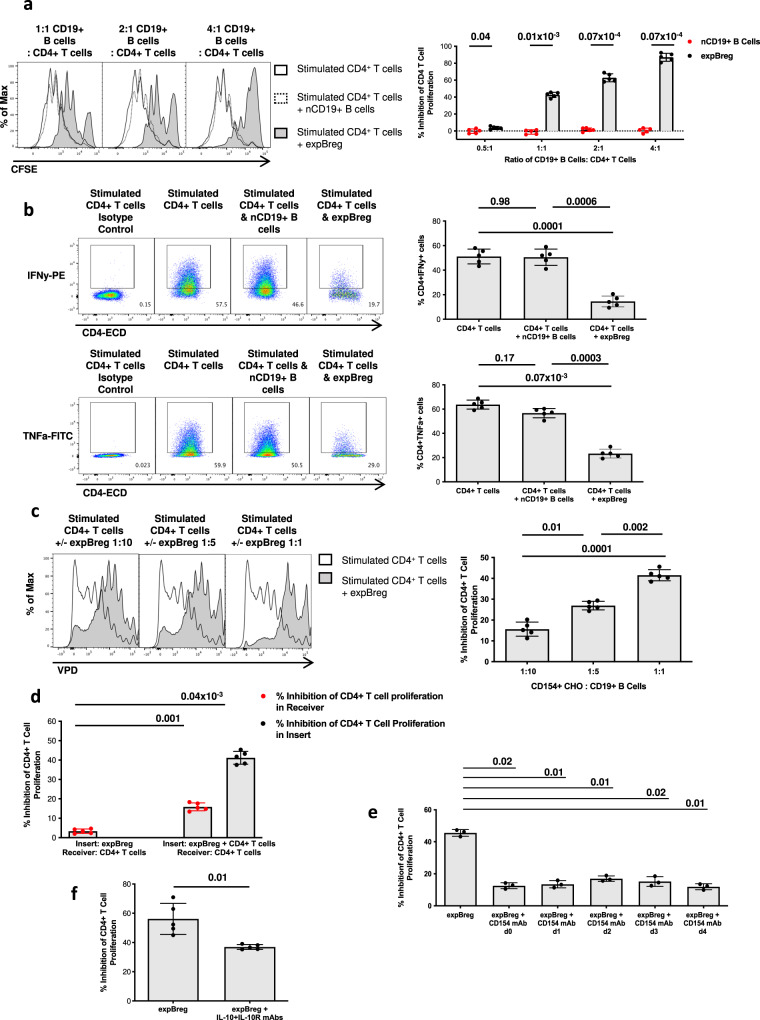
Expanded human CD19^+^IL-10^+^ B cells can suppress autologous CD4^+^ T-cell responses in vitro. **a** Representative histograms of live CD4^+^CFSE^+^ T cells and summarised data are shown. Autologous CD4^+^CFSE^+^ T cells were cultured with anti-CD3/CD28 beads for 5 days ± expBreg or nCD19^+^ B cells, at increasing ratios of CD19^+^ B cells to CD4^+^ T cells. % Inhibition of CD4^+^ T-cell proliferation is an expression of Division Index of live CD4^+^CFSE^+^ T cells at day 5 relative to that of the Stimulated CD4^+^ T-cell control. Data from experiments performed with cells from different healthy donors (*n* = 5) are presented. Each dot is an individual response. Representative FACS plots of CD4^+^CFSE^+^ T cells are shown in Supplementary Fig. 2a. **b** Representative FACS plots and summarised data of IFNy and TNFa expression by human CD4^+^ T cells which were cultured with anti-CD3/CD28 beads for 3 days ± expBreg or nCD19^+^ B cells at increasing ratios of CD19^+^ B cells to CD4^+^ T cells, are shown. Gating is on Isotype controls. Data from experiments performed with cells from different healthy donors (*n* = 5) are presented. Each dot is an individual response. **c** Representative histograms of live CD4^+^VPD^+^ T cells and summarised data are shown when autologous CD4^+^VPD^+^ T cells were cultured with anti-CD3/CD28 beads for 5 days ± expBreg that had been expanded for 7 days at increasing ratios of CD154^+^ CHO cells to CD19^+^ B cells. Data from experiments performed with cells from different healthy donors (*n* = 5) are presented. Each dot is an individual response. **d** Summarised data of % inhibition of CD4^+^ T-cell proliferation by expBreg within transwell assays. ‘Receiver’ refers to the bottom receiver well of the transwell system whilst ‘Insert’ refers to the transwell insert. Data from experiments performed with cells from different healthy donors (*n* = 5) are presented. Each dot is an individual response. **e** Summarised data of % inhibition of CD4^+^ T-cell proliferation by expBreg when in the presence of blocking CD154 mAb. Blocking CD154 mAb was added between day-1 and day-4 of the 5-day suppression assay. Data from experiments performed with cells from different healthy donors (*n* = 3) are presented. Each dot is an individual response. **f** Summarised data of % inhibition of CD4^+^ T-cell proliferation by expBreg when in the presence of blocking IL-10 and IL-10R mAbs. Blocking mAbs were added at day-0 of the 5-day suppression assay. Data from experiments performed with cells from different healthy donors (*n* = 5) are presented. Each dot is an individual response. Error bars in each panel represent Mean ± SD (**a**–**f**). One-way ANOVA with Tukey’s multiple comparisons test (**b**–**e**) and 2-tailed paired *t* test (**a**, **f**) were used. Source data are provided as a Source Data file.

Whilst a substantial proportion of expanded human CD19^+^ B cells produced the immunosuppressive cytokine IL-10, it was critical to establish whether this B-cell population could exert regulatory function in order to fulfil the definition of a human regulatory B cell. We co-cultured CD19^+^ B cells expanded at a 1:1 ratio with CD154^+^ CHO cells as described above, with autologous, human CD4^+^ T cells which were stimulated with anti-CD3/CD28 beads. Expanded human CD19^+^ B cells (expBreg) suppressed autologous CD4^+^ T-cell proliferation in a dose-dependent manner in vitro (Fig. 2a and Supplementary Fig. 2a). expBreg also suppressed the expression of pro-inflammatory cytokines interferon-γ (IFNγ) and tumour necrosis factor α (TNFα) by CD4^+^ T cells (Fig. 2b). expBreg which had been expanded at 1:1 ratio of CD154^+^ CHO cells to CD19^+^ B cells, were more suppressive than those expanded at lower ratios (Fig. 2c) despite no difference in IL-10 expression or production levels (Fig. 1b, c). This suggested that high levels of CD154-mediated B-cell stimulation were important to suppressive potency. In keeping with these findings, varying the cytokine stimulation within expansion co-cultures had no effect on the in vitro suppressive potency of expBreg (Supplementary Fig. 2b). Moreover, 7-day stimulation of human CD19^+^ B cells with agonistic CD40 mAb, known to provide weaker stimulation than membrane-bound ligand^24–26^, did not result in the generation of B cells with regulatory function (Supplementary Fig. 2c). Non-expanded CD19^+^ B cells (nCD19^+^ B cells) were unable to suppress CD4^+^ T-cell proliferation or inflammatory cytokine expression in vitro (Fig. 2a, b).

In keeping with endogenous human and mouse Breg studies^16,20,27^, transwell assays demonstrated that cognate interaction of expBreg with stimulated human CD4^+^ T cells was crucial to the suppressive function of this ex vivo-expanded human Breg population (Fig. 2d). When expBreg were cultured alone in the transwell insert and CD4^+^ T cells were stimulated by anti-CD3 and anti-CD28 beads in the receiver well, suppression of CD4^+^ T-cell proliferation was markedly reduced. However, if expBreg, human CD4^+^ T cells and anti-CD3 and anti-CD28 beads were co-cultured in the transwell insert, and additional CD4^+^ T cells and beads were cultured in the receiver well, not only was proliferation of the CD4^+^ T cells in the insert suppressed, but inhibition of CD4^+^ T-cell proliferation in the receiver well was also observed. These findings indicated that cognate interactions between expBreg and CD4^+^ T cells resulted in the release of a soluble factor that partially suppressed the CD4^+^ T-cell response. Blockade of CD154 or CD40 within the suppression assay resulted in abrogation of expBreg-mediated suppression, suggesting that this cognate interaction drove suppression of autologous CD4^+^ T cells by expBreg (Fig. 2e and Supplementary Fig. 2d). As expected, a proportion of human CD4^+^ T cells upregulated CD154 expression within 48 h of activation by anti-CD3 and anti-CD28 beads within the suppression assay (Supplementary Fig. 2d). Suppression of CD4^+^ T-cell proliferation in the presence of expBreg was observed from 72 h of the suppression assay (Supplementary Fig. 2e). Constant interaction between CD154 and CD40 was required for expBreg-mediated suppression, as CD4^+^ T-cell proliferation recovered when anti-CD154 mAb was added as late as day 4 of a 5-day suppression assay, despite significant suppression having already occurred by this point (Fig. 2e). Thus similar to the observation that repeated CD154-mediated stimulation was required to maintain expBreg in expansion co-cultures for 14 days (Fig. 1e), persistent CD154–CD40 interaction was required within suppression assays to maintain expBreg suppressive function.

In keeping with the majority of endogenous human Breg populations^3,4,21^, expBreg demonstrated possible dependence on IL-10 to exert regulatory function, as mAb blockade of IL-10 and IL-10R reduced suppressive potency of expBreg (Fig. 2f). This effect however, appeared to be only partial within in vitro suppression assays, suggesting that suppressive mechanisms in addition to IL-10 may be involved in the expBreg-mediated regulation of CD4^+^ T-cell responses. Other soluble suppressive factors reported to be secreted by endogenous Breg subsets, such as TGFβ and IL-35^9,28,29^, were not involved in expBreg-mediated suppression in vitro (Supplementary Fig. 2f, g).

Thus human CD19^+^ B cells could be cultured and maintained ex vivo to generate large numbers of B cells with in vitro regulatory function. Similar to endogenous human IL-10^+^ Breg populations, expBreg-mediated regulation of human CD4^+^ T-cell responses was dependent on cognate interactions and was driven by CD154–CD40. expBreg however, exhibited only partial dependence on the expression of IL-10.

### expBreg resemble endogenous CD73^−^CD25^+^CD71^+^IL-10^+^ human Breg, but suppressive function does not correlate with IL-10 expression

**Fig. 3:**
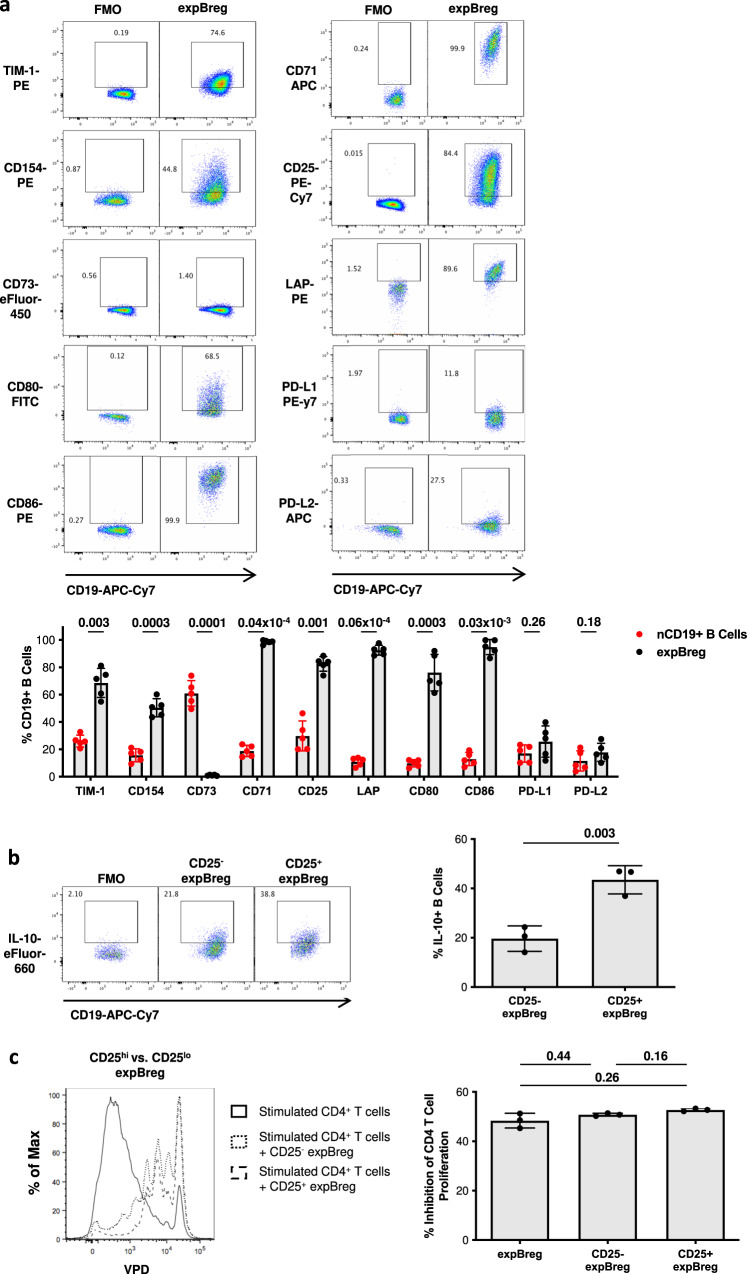
expBreg resemble endogenous CD73^−^CD25^+^CD71^+^IL-10^+^ Breg, but suppressive function does not correlate with IL-10 expression. **a** Representative FACS plots of live CD19^+^ B cells and summarised data demonstrating expression of cell-surface markers by expBreg. Gating is on FMO controls. % Expression is compared between expBreg and autologous non-expanded CD19^+^ B cells (nCD19^+^ B cells). Data from experiments performed with cells from different healthy donors (*n* = 5) are presented. Each dot is an individual response. Representative FACS plots of nCD19^+^ B cells are shown in Supplementary Fig. 3b. **b** Representative FACS plots of CD19^+^ expBreg and summarised data demonstrating expression of IL-10 are presented. Gating is on FMO controls. Data from experiments performed with cells from different healthy donors (*n* = 3) are presented. **c** Representative histograms of live CD4^+^VPD^+^ T cells and summarised data are presented when autologous CD4^+^VPD^+^ T cells and anti-CD3/CD28 beads were co-cultured alone or with CD25^+^ or CD25^−^ expBreg. Data from experiments performed with cells from different healthy donors (*n* = 3) are presented. Each dot is an individual response. Error bars in each panel represent Mean ± SD (**a**–**c**) 2-tailed paired *t* tests (**a**, **b**) and one-way ANOVA with Tukey’s multiple comparisons tests (**c**) were used. Source data are provided as a Source Data file.

To better understand whether expBreg had phenotypic characteristics consistent with endogenous human IL-10^+^ Breg populations, flow cytometric analysis of known Breg markers was performed^3,4,9,20,21,30–33^. expBreg upregulated expression of TIM-1, the putative Breg marker described in mice and in humans, as well as the activation marker CD154, following 7-day stimulation with CD154^+^ CHO cells (Fig. 3a)^22,34–37^. expBreg were predominantly CD73^−^CD25^+^CD71^+^, similar to endogenous human IL-10^+^ Breg identified by whole genome expression analysis^21^. A proportion of expBreg expressed LAP, the precursor to TGFβ, as well as CD80, CD86, PD-L1 and PD-L2, which have been linked to Breg-mediated suppressive function in earlier studies (Fig. 3a)^9,20,33^. expBreg did not upregulate other markers of mouse or human Breg such as CD1d, CD5, CD10, CD24, CD38 or CD27, when compared to nCD19^+^ B cells (Supplementary Fig. 3a, b).

Given that a proportion of expBreg-expressed IL-10 and that expBreg demonstrated partial dependence on IL-10 for suppressive function in vitro, phenotypic analysis of IL-10^+^ expBreg was also undertaken to elucidate potential functional and extracellular markers of suppressive function. CD25^+^ expBreg were enriched with IL-10^+^ B cells when compared to CD25^-^ expBreg (Fig. 3b), although CD25^−^ expBreg did demonstrate 19.7% ± 5.2 (mean ± SD) expression of IL-10. There was no enrichment of IL-10 expression with cell-surface markers of previously described human or mouse Breg subsets when compared to the respective negative populations (Supplementary Fig. 4a)^4,30–32,34–37^. TIM-1 expression was markedly down-regulated following PMA and Ionomycin stimulation (Supplementary Fig. 4a), but FACS-sorted TIM-1^+^ expBreg which were subsequently stimulated, were not enriched for IL-10^+^ B cells when compared to TIM-1^-^ expBreg (Supplementary Fig. 4b, c).

Despite the enrichment of IL-10 expression in CD25^+^ expBreg, there was no difference in suppressive potency between FACS-sorted CD25^+^ expBreg and CD25^−^ expBreg (Fig. 3c and Supplementary Fig. 4d). Thus, whilst expBreg have a phenotype consistent with an existing endogenous IL-10^+^ human Breg population, our observations suggest that IL-10 production may not the predominant mechanism by which expBreg suppress autologous CD4^+^ T-cell responses.

### Suppressive function of expBreg is dependent on expression of TIM-1 and CD154

**Fig. 4:**
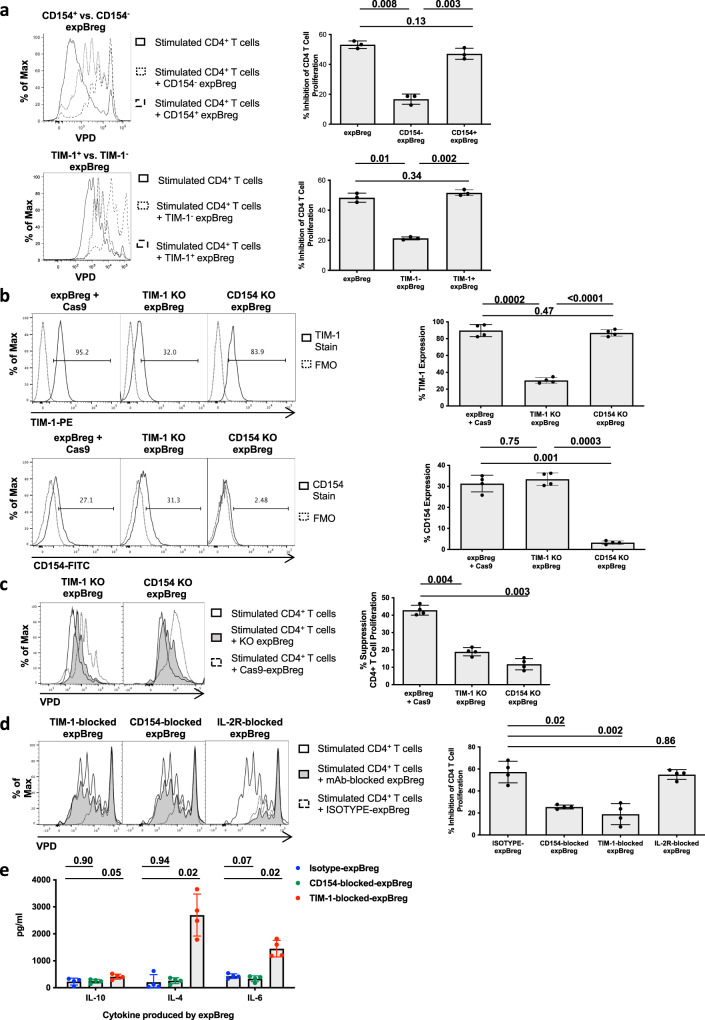
Suppressive function of expBreg is dependent on expression of TIM-1 and CD154. **a** Representative histograms of live CD4^+^VPD^+^ T cells and summarised data of % inhibition of CD4^+^ T-cell proliferation are presented when autologous CD4^+^VPD^+^ T cells and anti-CD3/CD28 beads were co-cultured alone or with CD154^+^, CD154^−^, TIM-1^+^ or TIM-1^−^ expBreg. Data from experiments performed with cells from different healthy donors (*n* = 3) are presented. Each dot is an individual response. **b** Representative histograms of live CD19^+^ expBreg and summarised data are presented which demonstrate protein expression of TIM-1 and CD154 after electroporation with Cas9 alone ± TIM-1 or CD154 multiguide RNAs to generate TIM-1 or CD154 knockouts (KO). Data from experiments performed with cells from different healthy donors (*n* = 4) are presented. Each dot is an individual response. **c** Representative histograms of live CD4^+^VPD^+^ T cells and summarised data of % inhibition of CD4^+^ T-cell proliferation are presented when autologous CD4^+^VPD^+^ T cells and anti-CD3/CD28 beads were co-cultured alone or with TIM-1 KO, CD154 KO or Cas9-expBreg. Data from experiments performed with cells from different healthy donors (*n* = 4) are presented. Each dot is an individual response. **d** Representative histograms of live CD4^+^VPD^+^ T cells and summarised data of % inhibition of CD4^+^ T-cell proliferation are presented when autologous CD4^+^VPD^+^ T cells and anti-CD3/CD28 beads were co-cultured alone or with expBreg which had been pre-incubated with IgG isotype control (Isotype-expBreg), anti-CD154 mAb (CD154-blocked-expBreg), anti-TIM-1 mAb (TIM-1-blocked-expBreg) or anti-IL-2R mAbs (IL-2R-blocked-expBreg) for the last 48 h of expansion. Data from experiments performed with cells from different healthy donors (*n* = 4) are presented. Each dot is an individual response. **e** Summarised data of human cytokines produced by Isotype-expBreg, CD154-blocked expBreg or TIM-1-blocked expBreg. Supernatants were analysed by cytokine bead array following a 5 h incubation period of expBreg at 37 °C in complete media immediately following expansion. Data from experiments performed with cells from different healthy donors (*n* = 4) are presented. Each dot is an individual response. Error bars in each panel represent Mean ± SD (**a**–**e**). One-way ANOVA with Tukey’s multiple comparisons tests were used (**a**–**e**). Source data are provided as a Source Data file.

To interrogate other mechanisms by which expBreg may exert suppressive function, FACS-isolation of expBreg populations or mAb-blockade was undertaken based on phenotypic markers which were upregulated following expansion (Fig. 3a). Suppressive function was evaluated in vitro. FACS-sorted TIM-1^+^ expBreg and CD154^+^ expBreg were more suppressive than TIM-1^−^ expBreg and CD154^−^ expBreg respectively (FACS purities 73–95%) (Fig. 4a and Supplementary Fig. 5a). There was no difference in suppressive function between FACS-isolated CD73^−^CD25^+^CD71^+^ and CD73^−^CD25^−^CD71^+^ expBreg populations (Supplementary Fig. 5b). However, when these two subsets were subsequently sorted based on TIM-1 or CD154 expression, suppressive function again correlated with the expression of TIM-1 and CD154, thus reinforcing the importance of these two markers in expBreg suppressive function (Supplementary Fig. 5c). mAb-blockade of CD80, CD86, PD-L1 or PD-L2 in suppression assays, did not abrogate suppressive function of expBreg, indicating no direct involvement of these markers (Supplementary Fig. 5d–f). Suppressive potency of expBreg was associated with the expression of TIM-1 and CD154, rather than the CD73^−^CD25^+^CD71^+^ phenotype or IL-10.

To clarify the functional roles of TIM-1 and CD154, we interrogated these extracellular markers using two different approaches. We first employed the CRISPR-Cas9 system as previously described^38^, to knock out TIM-1 or CD154 in expBreg. The use of multiguide RNAs and Cas9 protein to generate ribonucleotide proteins (RNPs), resulted in 66.1% ± 1.6 (mean ± SD) and 89.6% ± 2.5 (mean ± SD) loss of cell-surface TIM-1 and CD154 protein expression by expBreg respectively (Fig. 4b). Importantly, the expression of CD154 in TIM-1-knockout expBreg and the expression of TIM-1 in CD154-knockout expBreg remained unchanged when compared to control conditions (Fig. 4b), indicating the specificity of each knockout. TIM-1-knockout expBreg and CD154-knockout expBreg both exhibited reduced suppressive function when compared to expBreg undergoing electroporation alone ± Cas9 (Fig. 4c), demonstrating that both markers were required for expBreg-mediated suppression. The CD73^−^CD25^+^CD71^+^ phenotype was maintained in each knockout, and IL-10 production was unchanged (Supplementary Fig. 5g, h).

In an alternative approach to examine the functional roles of TIM-1 and CD154, expBreg were pre-incubated with blocking mAbs to either CD154 or TIM-1 before suppressive function was assessed in vitro^39^. expBreg which had been pre-incubated with a blocking mAb to either CD154 or TIM-1 subsequently displayed reduced suppressive function, again demonstrating a functional role for both expBreg-expressed CD154 and TIM-1 (Fig. 4d). Fluorescence-conjugated mAbs with the same epitope specificities as the blocking mAbs were utilised to phenotype pre-incubated expBreg and confirm effective antibody binding (Supplementary Fig. 5i)^39^. Similar to the CRISPR-Cas9 approach, the expression of TIM-1 in the presence of CD154-blockade, and the expression of CD154 in the presence of TIM-1-blockade, was maintained. Unlike the CRISPR-Cas9 approach, mAb-blockade of either CD154 or TIM-1 resulted in the downregulation of CD71 and upregulation of CD73 expression (Supplementary Fig. 5i). These findings may reflect artefactual differences between the two experimental techniques, or off-target effects of mAb-blockade. As with the CRISPR-Cas9 approach, IL-10 production by TIM-1-blocked expBreg and CD154-blocked expBreg remained unchanged when compared to control conditions (Fig. 4e). Xiao et al. found that mouse B cells with TIM-1 defects had impaired IL-10 production but increased pro-inflammatory cytokine production^37^. TIM-1-blockade in human expBreg similarly resulted in an increase in the production of pro-inflammatory cytokines IL-4 and IL-6 by expBreg, although the production of IL-10 remained unchanged (Fig. 4e). CD154-blockade did not affect pro-inflammatory cytokine or IL-10 production, indicating that this action of TIM-1 was independent of CD154.

Using two different experimental approaches, we demonstrate that suppressive potency of expBreg is dependent upon expression of both TIM-1 and CD154.

### Suppressive function of expBreg is dependent on TIM-1-mediated regulation of STAT3 phosphorylation

**Fig. 5:**
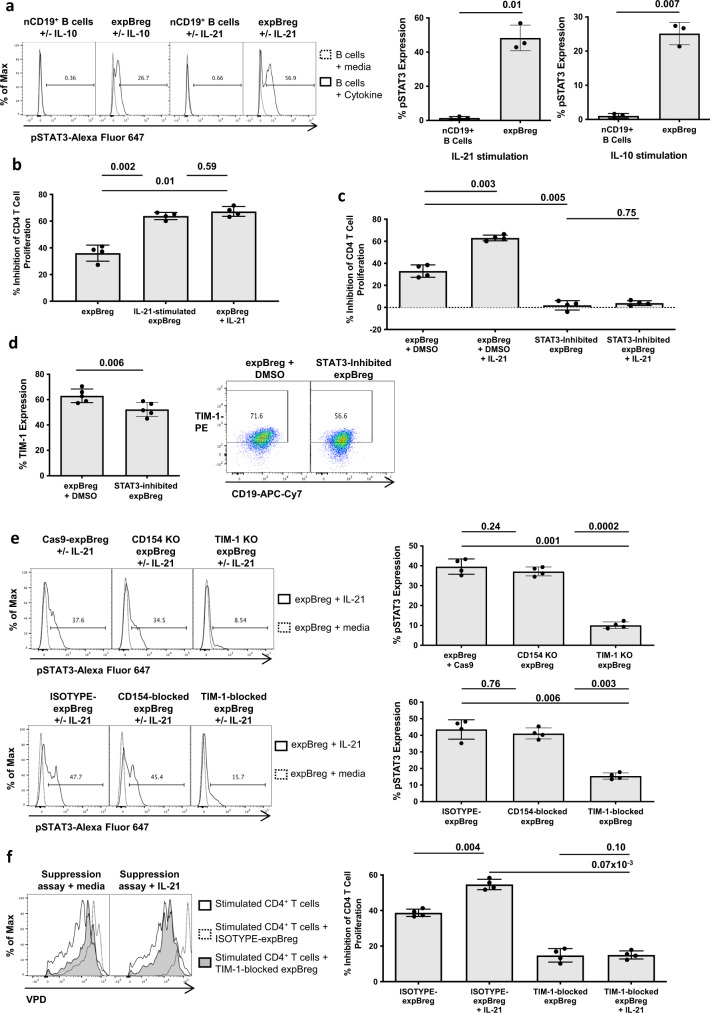
Suppressive function of expBreg is dependent on TIM-1-mediated regulation of STAT3 phosphorylation. **a** Representative histograms of CD19^+^ B cells and summarised data of % STAT3 phosphorylation (pSTAT3) are presented when non-expanded CD19^+^ B cells (nCD19^+^ B cells) or expBreg were incubated with media, IL-10 [0.01 μg/ml] or IL-21 [0.05 μg/ml] for 10 min and stained for pSTAT3. Data from experiments performed with cells from different healthy donors (*n* = 3) are presented. Each dot is an individual response. **b** Summarised data of % inhibition of CD4^+^ T-cell proliferation are presented when autologous CD4^+^CFSE^+^ T cells and anti-CD3/CD28 beads were co-cultured with expBreg ± IL-21. expBreg were either pre-incubated with IL-21 [0.05 μg/ml] for the last 48 h of the 7-day expansion co-culture (IL-21-stimulated expBreg) prior to addition to the suppression assay at day-0, or IL-21 [0.05 μg/ml] was directly added at day-0 of the suppression assay ±  expBreg (expBreg + hIL-21). Data from experiments performed with cells from different healthy donors (*n* = 4) are presented. Each dot is an individual response. Representative histograms of live CD4^+^CFSE^+^ T cells are presented in Supplementary Fig. 6c. **c** Summarised data of % inhibition of CD4^+^ T-cell proliferation are presented when autologous CD4^+^CFSE^+^ T cells and anti-CD3/CD28 beads were co-cultured with expBreg ± STAT3 inhibitor ± IL-21 [0.05 μg/ml]. expBreg were incubated for 2 h with the STAT3 inhibitor WP 1066 [10 μM] ± hIL-21 [0.05 μg/ml], or DMSO ± hIL-21 [0.05 μg/ml], washed and suppressive function analysed. Data from experiments performed with cells from different healthy donors (*n* = 4) are presented. Each dot is an individual response. Representative histograms of live CD4^+^CFSE^+^ T cells are presented in Supplementary Fig. 7a. **d** Representative FACS plots of live CD19^+^ expBreg and summarised data are presented which demonstrate expression of TIM-1 in the presence of STAT3 inhibition. Data from experiments performed with cells from different healthy donors (*n* = 5) are presented. Each dot is an individual response. **e** Representative histograms of CD19^+^ expBreg and summarised data of % STAT3 phosphorylation (pSTAT3) in response to IL-21 stimulation, are presented following gene knockouts or mAb blockade of TIM-1 or CD154 within expBreg. Data from experiments performed with cells from different healthy donors (*n* = 4) are presented. Each dot is an individual response. **f** Representative histograms of live CD4^+^VPD^+^ T cells and summarised data of % inhibition of CD4^+^ T-cell proliferation are presented when autologous CD4^+^VPD^+^ T cells and anti-CD3/CD28 beads were co-cultured alone or with mAb-blocked expBreg ± IL-21 [0.05 μg/ml]. expBreg had been pre-incubated with IgG isotype control (Isotype-expBreg) or anti-TIM-1 mAb (TIM-1-blocked-expBreg) for the last 48 h of expansion. Data from experiments performed with cells from different healthy donors (*n* = 4) are presented. Each dot is an individual response. Error bars in each panel represent Mean ± SD (**a**–**f**). 2-tailed paired *t* tests (**a**, **d**) and one-way ANOVA with Tukey’s multiple comparisons tests (**b**, **c**, **e**, **f**) were used. Source data are provided as a Source Data file.

The STAT3 signalling pathway has been associated with regulatory function of endogenous human IL-10^+^ Breg in the context of transplantation, GVHD and autoimmune disease^3,40–43^. We asked whether similarly, suppressive function of expBreg was associated with STAT3 signalling. expBreg which had been rested for 1.5 h after expansion, expressed pSTAT3 in response to a 10 min incubation period with exogenous IL-10 [0.01 μg/ml] or IL-21 [0.05 μg/ml], unlike non-expanded CD19^+^ B cells (Fig. 5a). Surprisingly, despite expBreg expressing the IL-6 receptor (IL-6R) (Supplementary Fig. 6a), exogenous IL-6 did not stimulate STAT3 phosphorylation in expBreg, even at concentrations up to 10 μg/ml (Supplementary Fig. 6b).

IL-21 is known to induce mouse B10-cell expansion and drive IL-10 production^16,44^. Stimulation of expBreg with IL-21 [0.05 μg/ml] for the last 48 h of the 7-day expansion culture (IL-21-stimulated expBreg), or direct addition of IL-21 [0.05 μg/ml] to the suppression assay at day-0 (expBreg + Il-21), resulted in increased suppressive potency (Fig. 5b and Supplementary Fig. 6c). Similar exposure of expBreg to IL-6 during the expansion culture did not affect expBreg suppressive function (Supplementary Fig. 6d). Expression of CD40, CD154 or TIM-1 and production of IL-10 by expBreg stimulated with IL-21 remained unchanged (Supplementary Fig. 6e, f). Of note, proliferation of expBreg did not increase when exposed to IL-21 within expansion co-cultures or suppression assays (Supplementary Fig. 6g, h). When pre-incubated with a STAT3 inhibitor, expBreg were unable to suppress CD4^+^ T-cell proliferation, demonstrating the importance of this signalling pathway within human expBreg (Fig. 5c and Supplementary Fig. 7a). Whilst there was a slight downregulation of TIM-1 expression in the presence of STAT3-inhibition (Fig. 5d), the expression of CD40 and CD154, and production of IL-10 were unchanged (Supplementary Fig. 7b, c). Crucially, expBreg function could not be rescued by pre-incubation with IL-21 when in the presence of STAT3 inhibition, indicating that it is through this intracellular pathway that IL-21 promotes suppressive function by expBreg (Fig. 5c and Supplementary Fig. 7a).

These data demonstrate that expBreg-mediated suppression was directly dependent upon STAT3 phosphorylation. The suppressive potency of human expBreg cells could be enhanced by increasing levels of STAT3 phosphorylation, such as by cytokine stimulation with IL-21. The stimulus for STAT3 phosphorylation appears to be tightly controlled, such that other known inducers of pSTAT3, such as IL-6, were unable to trigger STAT3 phosphorylation and affect expBreg function, despite high levels of IL-6R expression by expBreg.

We asked whether TIM-1 or CD154 was involved in this signalling pathway. Both TIM-1-knockout expBreg and TIM-1-blocked expBreg demonstrated reduced levels of STAT3 phosphorylation in response to stimulation by IL-21 (Fig. 5e). Neither CD154-knockout nor CD154-blockade affected STAT3 phosphorylation within expBreg (Fig. 5e). The upregulation of pSTAT5 in response to IL-2 or IL-4^44–47^ was unchanged in the presence of either TIM-1-knockout or TIM-1 blockade (Supplementary Fig. 7d), demonstrating the specific effect of TIM-1 upon STAT3 phosphorylation. In keeping with these observations, exposure of expBreg to high concentrations of IL-2 during the suppression assay did not affect expBreg suppressive function (Supplementary Fig. 7e). Importantly, the addition of recombinant IL-21 to suppression co-cultures could not rescue expBreg suppressive function in the presence of TIM-1-blockade (Fig. 5f). Thus TIM-1 modulates the phosphorylation of STAT3, and in doing so regulates the suppressive function of the expBreg cell.

### expBreg can prolong allograft survival in a humanised mouse model of human skin transplantation

**Fig. 6:**
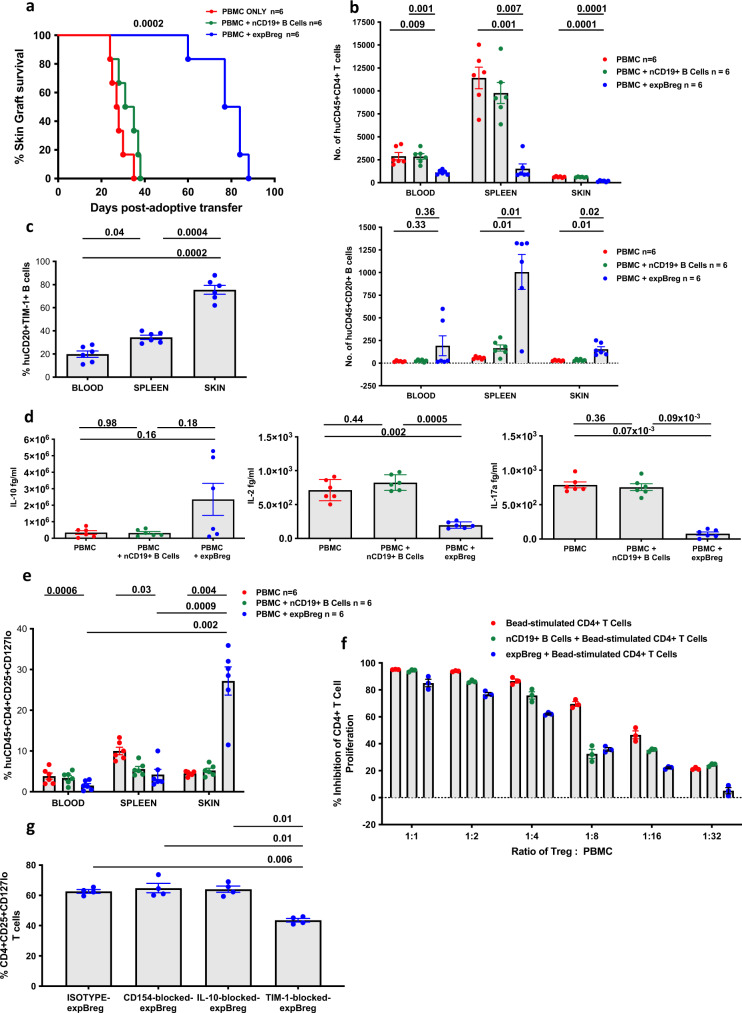
expBreg can prolong allograft survival in a humanised mouse model of human skin transplantation. **a** Kaplan–Meier survival curve demonstrating human allograft survival in a humanised mouse model of skin transplantation. Balb/c Rag2^−/−^ cy^−/−^ mice which were transplanted with human skin allograft are reconstituted with human PBMC alone or ±non-expanded CD19^+^ B cells or expBreg from a different human donor. *n* = 6 per group per experiment, one of three independent experiments is shown. Experimental schematic is presented in Supplementary Fig. 8a. **b** Summarised data of number of huCD45^+^CD4^+^ T cells and huCD45^+^CD20^+^ B cells of mice. *n* = 6 per group per experiment, one of three independent experiments is shown. Each dot is an individual mouse response. Gating strategy and representative histograms of live huCD45^+^ cells are presented in Supplementary Fig. 8b. **c** Summarised data of % expression of huCD45^+^CD20^+^TIM-1^+^ B cells in mice receiving PBMC + expBreg. *n* = 6 per group per experiment, one of three independent experiments is shown. Each dot is an individual mouse response. Representative FACS plots of live huCD45^+^CD20^+^TIM-1^+^ B cells are presented in Supplementary Fig. 8d. **d** Summarised data of serum levels of human IL-10, IL-2 and IL-17a in blood of mice. *n* = 6 per group per experiment, one of three independent experiments is shown. Each dot is an individual mouse response. **e** Summarised data of % live huCD45^+^CD4^+^CD25^+^CD127^lo^ Treg of mice. *n* = 6 per group per experiment, one of three independent experiments is shown. Each dot is an individual mouse response. Representative FACS plots of live huCD45^+^CD4^+^CD25^+^CD127^lo^ T cells are presented in Supplementary Fig. 8f. **f** Summarised data of % inhibition of CD4^+^ T-cell proliferation by human CD4^+^CD25^+^CD127^lo^ Treg which were generated in the presence or absence of expBreg. Data from experiments performed with cells from different healthy donors (*n* = 3) are presented. Each dot is an individual response. Representative FACS plots of live CD4^+^CD25^+^CD127^lo^ T cells are shown in Supplementary Fig. 8g. **g** Summarised data of % live CD4^+^CD25^+^CD127^lo^ human Treg on day-5 of suppression assay when autologous CD4^+^ T cells were co-cultured with anti-CD3/CD28 beads ± Isotype-expBreg, CD154-blocked-expBreg, IL-10-blocked-expBreg or TIM-blocked-expBreg. Data from experiments performed with cells from different healthy donors (*n* = 4) are presented. Each dot is an individual response. In (**a**–**e**), each experiment used a different HLA-mismatched human donor pair. Error bars in (**b**–**e**) represent Mean ± SEM. Error bars in (**f**, **g**) represent Mean ± SD. Log-rank (Mantel–Cox) test (**a**) and one-way ANOVA with Tukey’s multiple comparisons tests (**b**–**e**, **g**) were used. Source data are provided as a Source Data file.

To determine whether this ex vivo-expanded human Breg population maintained regulatory function in vivo, we utilised a humanised mouse model of human skin transplantation^48,49^. Transplanted mice receiving PBMC and expBreg demonstrated prolonged allograft survival when compared to those that had received PBMC alone or PBMC and nCD19^+^ B cells (Fig. 6a and Supplementary Fig. 8a). FACS analysis demonstrated a significant decrease in the number of huCD45^+^CD4^+^ T cells and a significant increase in the number of huCD45^+^CD20^+^ B cells in both the human skin allograft and spleen of mice which had been reconstituted by PBMC and expBreg, in comparison to control groups (Fig. 6b and Supplementary Fig. 8b). There was no difference in the number of huCD45^+^CD8^+^ T cells between experimental groups (Supplementary Fig. 8b, c). Within the skin allografts of mice that had received PBMC and expBreg, huCD45^+^CD20^+^ B cells were predominantly TIM-1^+^, with lower levels of TIM-1 expression in blood and spleen (Fig. 6c and Supplementary Fig. 8d). Analysis of peripheral blood demonstrated no differences in serum levels of human immunoglobulin or hIL-10 across groups (Fig. 6d and Supplementary Fig. 8e). However, levels of pro-inflammatory cytokines hIL-2 and hIL-17a were significantly lower in mice that had received PBMC and expBreg (Fig. 6d). To our knowledge, this is the first demonstration of in vivo regulatory function of a human Breg population.

There is growing evidence that Breg may support the induction and expansion of Treg^6,9,20,29,50^. We found an almost threefold increase in percentage of putative huCD45^+^CD4^+^CD25^+^CD127^lo^ Treg within the skin allografts of mice reconstituted with PBMC and expBreg, compared to control groups (Fig. 6e and Supplementary Fig. 8f). The percentage of both huCD45^+^CD20^+^TIM-1^+^ Breg and huCD45^+^CD4^+^CD25^+^CD127^lo^ Treg were enriched within human skin allografts when compared to spleen and blood of mice that had received PBMC and expBreg, unlike in control groups (Fig. 6c, e). These observations suggest an immunoregulatory-rich environment within the allograft that may promote increased graft survival, when humanised mice are reconstituted with expBreg. Although an insufficient number of Treg could be extracted from human skin allografts to test function (Supplementary Fig. 8f), in vitro experiments confirmed that expBreg were able to induce and expand functionally suppressive human CD4^+^CD25^+^CD127^lo^ Treg (Fig. 6f and Supplementary Fig. 8g). CD4^+^ T cells or CD4^+^CD25^−^ T cells from healthy human donors were stimulated for 5 days with anti-CD3 and anti-CD28 beads ± autologous nCD19^+^ B cells or expBreg. Percentage CD4^+^CD25^+^CD127^lo^ Treg was increased by day-5 when expBreg were co-cultured with autologous CD4^+^ T cells, compared to control conditions (Supplementary Fig. 8g). On day-5 of co-culture, CD4^+^CD25^+^CD127^lo^ Treg were FACS-isolated from each condition (>94% purity) and suppressive function was confirmed in suppression assays (Fig. 6f). TIM-1-blockade resulted in a decrease in frequency of expBreg-induced human Treg in vitro, whilst IL-10-blockade and CD154-blockade had no such effect (Fig. 6g). These findings indicate an additional mechanism by which TIM-1 may promote Breg-mediated suppression, and corroborate earlier work^37^. Thus the balance of T effectors (Teff) to Treg shifts in the presence of expBreg, such that we see an increase in the proportion of putative Treg and TIM-1^+^ Breg in human allografts with prolonged survival.

### CD154-dependent endogenous CD19^+^TIM-1^+^ Breg are enriched in blood and tumours of patients with cutaneous squamous cell carcinoma

**Fig. 7:**
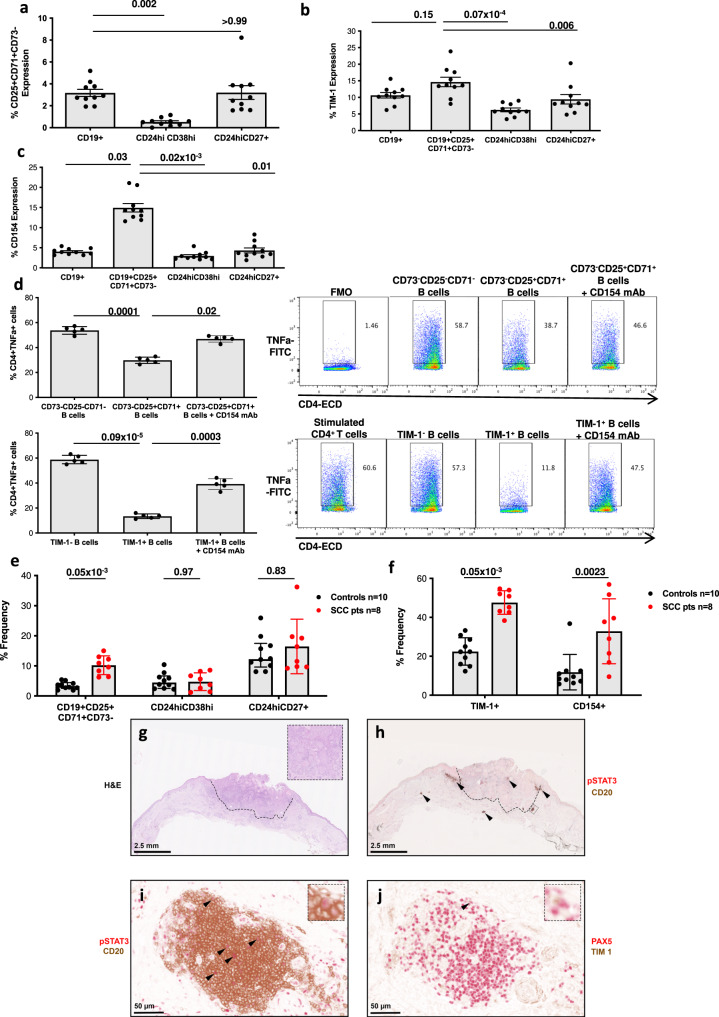
CD154-dependent endogenous CD19^+^TIM-1^+^ Breg are enriched in blood and tumours of patients with cutaneous squamous cell carcinoma. **a** Summarised data of % CD73^-^CD25^+^CD71^+^ B cells in CD19^+^ B-cell subsets within peripheral blood of healthy human donors (*n* = 10). Each dot is an individual response. Representative FACS plots are presented in Supplementary Fig. 9a, b. **b** Summarised data of % TIM-1^+^ B cells in CD19^+^ B-cell subsets within peripheral blood of healthy human donors (*n* = 10). Each dot is an individual response. Representative FACS plots are presented in Supplementary Fig. 9c. **c** Summarised data of % CD154^+^ B cells in CD19^+^ B cell subsets within peripheral blood of healthy human donors (*n* = 10). Each dot is an individual response. Representative FACS plots are presented in Supplementary Fig. 9d. **d** Representative FACS plots and summarised data of % CD4^+^TNFa^+^ expression when FACS-sorted CD73^−^CD25^+^CD71^+^ enriched, CD73^−^CD25^−^CD71^−^, TIM-1^hi^ and TIM-1^lo^ B cells were cultured with autologous CD4^+^ T cells and plate-bound anti-CD3 mAb [0.5 µg/ml] ± blocking anti-CD154 mAb [10 µg/ml] for 3 days. Gating is on FMO controls. Data from experiments performed with cells from different healthy donors (*n* = 5) are presented. Each dot is an individual response. **e** Summarised data of % CD73^−^CD25^+^CD71^+^, CD24^hi^CD38^hi^ and CD24^hi^CD27^+^ B cell subsets in whole CD19^+^ B cells in peripheral blood of healthy human donors (*n* = 10) and age-matched patients with squamous cell carcinoma of the skin (SCC, *n* = 8). Each dot is an individual response. **f** Summarised data of % TIM-1^+^ and CD154^+^ B cells within CD73^−^CD25^+^CD71^+^ B cell subsets in peripheral blood of healthy human donors (*n* = 10) and age-matched patients with SCC (*n* = 8). Each dot is an individual response. **g** Hematoxylin and eosin (H&E) stained histological section demonstrating a moderately differentiated cutaneous SCC arising on the dorsum of the right hand of an 81-year-old male (hashed line delineates tumour boundary; higher magnification image demonstrating representative histological features in top right-hand corner). **h** Double immunohistochemistry staining for pSTAT3 (red) and CD20 (brown) demonstrates the peritumoral accumulation of CD20^+^ B-cell clusters (arrows) within the tumour and in proximity to its invasive margin (hashed line). **i** Higher magnification of the annotated B-cell cluster in (**h**) (hashed box), demonstrating double positive cells (arrows). **j** On adjacent section, a single cell double stained for PAX5 (red) and TIM-1 (brown) is identified (arrow; higher magnification image of cell in top right-hand corner). Histological sections in (**g**–**j**) are of an SCC tumour from one of five patients. Histological SCC sections of remaining four patients are presented in Supplementary Fig. 10a–d. Error bars in (**a**–**c**, **e**, **f**) represent Median + interquartile range. Error bars in (**d**) represent Mean ± SD. One-way ANOVA with Dunn’s multiple comparisons test (**a**–**d**) and 2-tailed Mann–Whitney test (**e**, **f**) were used. Source data are provided as a Source Data file.

We have established that expBreg express the CD73^−^CD25^+^CD71^+^ phenotype similar to an endogenous human IL-10^+^ Breg population, and that suppressive function of expBreg is dependent on TIM-1 and CD154. We asked whether there existed an endogenous human Breg population with similar suppressive properties to expBreg in healthy human donors. Phenotypic analysis of PBMC isolated from peripheral blood of 10 healthy individuals identified CD73^−^CD25^+^CD71^+^ B cells within endogenous CD19^+^ B cells, including the memory CD24^hi^CD27^+^ and transitional CD24^hi^CD38^hi^ B cell subsets known to be enriched for IL-10^+^ Breg (Fig. 7a and Supplementary Fig. 9a, b)^3,4^. In keeping with previous reports (^3,4,21^, all three Breg subsets were enriched with IL-10^+^ B cells (Supplementary Fig. 9b). Endogenous CD73^−^CD25^+^CD71^+^ B cells in human peripheral blood were enriched for CD154 and TIM-1 when compared to whole CD19^+^ B cells, CD24^hi^CD27^+^ or CD24^hi^CD38^hi^ B cell populations (Fig. 7b, c and Supplementary Fig. 9c, d). Endogenous CD73^-^CD25^+^CD71^+^ enriched B cells (Supplementary Fig. 9e) were able to suppress the expression of pro-inflammatory cytokine TNFα by CD4^+^ T cells when stimulated by plate-bound anti-CD3 mAb by a CD154-dependent mechanism (Fig. 7d), similar to expBreg. Whilst it was not possible to FACS-sort endogenous CD73^−^CD25^+^CD71^+^TIM-1^+^ B cells to sufficient purity and number, we did find that endogenous TIM-1^+^ B cells were also able to suppress expression of TNFα by CD4^+^ T cells in a CD154-dependent mechanism, suggesting concordancy between CD73^−^CD25^+^CD71^+^ Breg and TIM-1^+^ Breg (Fig. 7d and Supplementary Fig. 9e).

Several animal models have demonstrated the role of B cells in the promotion of tumour development, in particular SCC^51–53^. We found that the frequency of CD73^−^CD25^+^CD71^+^ Breg in circulating blood of patients diagnosed with cutaneous SCC was increased when compared to that of age-matched healthy controls, unlike CD24^hi^CD38^hi^ and CD24^hi^CD27^+^ B cell subsets (Fig. 7e). Importantly, the expression of TIM-1 and CD154 was enriched within CD73^−^CD25^+^CD71^+^ Breg of SCC patients in comparison to CD73^−^CD25^+^CD71^+^ Breg of healthy controls (Fig. 7f). Whilst there was an increase in TIM-1 expression in whole CD19^+^ B cells of patients with SCC, there was no difference in CD154 expression (Supplementary Fig. 9f). There was no difference in IL-10 expression by CD73^−^CD25^+^CD71^+^ Breg in the peripheral blood of patients with SCC when compared to controls (Supplementary Fig. 9g). Immunohistochemical analysis of five human primary cutaneous invasive SCCs demonstrated the peritumoral accumulation of CD20^+^ B cells, predominantly arranged as clusters, which were located both within the tumours themselves and in proximity to their invasive margins (Fig. 7g, h). Importantly, double chromogenic staining revealed the presence of both CD20^+^pSTAT3^+^ B cells and PAX5^+^TIM-1^+^ B cells in all five cases (Fig. 7i, j and Supplementary Fig. 10a–d).

Thus, it may be that the increased frequency of TIM-1^+^ Breg in the peripheral blood of patients with a newly diagnosed cancer is indicative of an over-regulated immune system. The presence of B-cell clusters demonstrating both TIM-1 and pSTAT3 expression within human cutaneous SCCs supports this hypothesis. Mouse B cells have been shown to promote tumour progression by promoting angiogenesis in a STAT3-dependent mechanism, although TIM-1 expression was not evaluated in this population^42^. In view of our findings, there may be homology between this human Breg population and mouse tumour-infiltrating, STAT3-dependent B cells identified in such studies.

## Discussion

Human IL-10^+^ Breg have been identified as enriched within several human B cell subsets present in peripheral blood^3,4,21,22,54^. However, human IL-10^+^ Breg populations are rare. In-depth characterisation is challenging, whilst the potential for targeting human IL-10^+^ Breg to augment or inhibit their function in vivo and their use as a cellular therapy has been limited. Here, we demonstrate that large numbers of human CD19^+^IL-10^+^ Breg could be generated, expanded and maintained for up to 14 days ex vivo by CD154 stimulation of whole human CD19^+^ B cell populations when isolated from peripheral blood. Whilst CD154-mediated activation of whole human CD19^+^ B cell populations has been used to generate B cells with regulatory function^20^, the expansion and maintenance over time of large numbers of human CD19^+^ Breg has not been described. This expanded human IL-10^+^ Breg population phenotypically resembled endogenous CD73^−^CD25^+^CD71^+^IL-10^+^ human Breg^21^, and also expressed the putative Breg marker, TIM-1. The ex vivo generation of large numbers of human IL-10^+^ Breg enabled the interrogation of regulatory function in vivo in a clinically relevant humanised mouse model of skin transplantation^48^. Adoptive transfer of human expBreg prolonged human allograft survival when compared to PBMC alone or PBMC and nCD19^+^ B cells. huCD45^+^CD20^+^TIM-1^+^ B cells not only persisted in mice which had received expBreg cells but were also enriched within the human allograft together with huCD45^+^CD4^+^CD25^+^CD127^lo^ Treg. This study provides proof of concept data that human Breg are able to suppress a human alloimmune response in vivo. Gene knockouts and mAb blockade demonstrate the dependence of expBreg upon TIM-1 and CD154 expression to mediate suppressive function. We describe a role for TIM-1 in the positive regulation of STAT3 phosphorylation within this ex vivo generated human Breg population.

Whilst expBreg demonstrated some dependence on IL-10 to exert suppressive function in vitro, IL-10-blockade resulted in only partial reduction of suppressive function. Importantly, gene knockout and mAb-blockade of either TIM-1 or CD154 resulted in markedly reduced suppressive function of expBreg, with no change in IL-10 production, indicating the involvement of IL-10-independent suppressive mechanisms. Much like other regulatory cell populations such as regulatory T cells (Treg), it may be that expBreg utilise multiple mechanisms to suppress immune responses. Whilst previously reported IL-10-independent mechanisms associated with Breg such as PD-1, TGFβ and IL-35 did not appear to be involved, alternative downstream pathways merit further exploration.

Downstream signalling of CD40 ligation in human Breg can be measured by monitoring the STAT3 cascade. Impaired STAT3 signalling is associated with reduced IL-10 production and impaired regulatory function by human CD24^hi^CD38^hi^ Breg in patients with systemic lupus erythematosus (SLE)^3^. The deletion of STAT3 in mouse B cells resulted in exacerbated experimental autoimmune uveitis, reduced murine IL-10^+^ and IL-35^+^ Breg frequency and reduced Treg expansion^55^. expBreg-mediated suppressive function was also dependent upon STAT3 phosphorylation. STAT3-inhibition abrogated regulatory function of human expBreg, and promotion of STAT3 phosphorylation by IL-21 stimulation increased suppressive potency. IL-10 production was unchanged, again indicating that mechanisms independent of IL-10 were predominant in exerting expBreg-mediated suppressive function. Surprisingly, an alternative trigger of the STAT3 cascade, IL-6, was unable to promote expBreg function nor induce STAT3 phosphorylation, despite cell-surface expression of IL-6R. This is contrary to observations in mouse Breg, where pro-inflammatory cytokines IL-6 and IL-1b have been reported to induce murine IL-10^+^ Breg^10^. These findings not only highlight the heterogeneity between induced mouse and human Breg, but also suggest tightly regulated signalling pathways within the STAT3 cascade.

We describe a mechanism by which TIM-1 acts as a gatekeeper to control human Breg function through the modulation of STAT3 phosphorylation. TIM-1 also appears to inhibit the production of pro-inflammatory cytokines by expBreg, maintaining regulatory function. In addition, expBreg may alter the balance of Teff and Treg to promote a more immunosuppressive environment in vivo, in a partly TIM-1-dependent mechanism. The control of STAT3 phosphorylation by TIM-1 could account for such broad-ranging effects. A slight downregulation in TIM-1 expression by expBreg was observed upon STAT3-inhibition, whilst expression of CD40 and CD154 remained unchanged. STAT3 has been reported to bind to the TIM-1 promoter and induce TIM-1 mRNA and protein expression^56^. The elucidation of potential feedback pathways involving TIM-1 and the STAT3 cascade may be important in better understanding the mechanisms which induce and regulate human Breg suppressive function.

It is not evident from this work how TIM-1 positively regulates STAT3 phosphorylation to dictate human Breg suppressive function. Nor is it understood how expBreg exert suppressive function downstream of TIM-1 and STAT3 signalling, or of CD154. One could speculate on the involvement of other regulators within the JAK/STAT pathway, such as Suppressor of Cytokine Signalling (SOCS) proteins, but this will warrant further investigation and is beyond the scope of the current study.

The in vivo regulation of human alloimmune responses to prolong the survival of human allograft suggests that the function of expBreg is stable over time in a complex biological environment. This is reinforced not only by the identification of human CD45^+^CD20^+^TIM-1^+^ B cells within both peripheral blood and spleen but also within the human allograft. The observations that human CD4^+^CD25^+^CD127^lo^ Treg were enriched in mice which received human expBreg, and that expBreg could promote human CD4^+^CD25^+^CD127^lo^ Treg induction in vitro, suggest an additional mechanism by which expBreg may exert suppressive function. Recent work has demonstrated the importance of the balance between human IL-10^+^ Breg and B effector cells in the form of an IL-10:TNFα ratio, to accurately predict acute rejection and allograft dysfunction in human kidney transplant recipients^11^. The frequency of endogenous CD19^+^TIM-1^+^ and CD19^+^CD154^+^ B cells in peripheral blood may serve as additional regulatory markers of alloimmunity in transplantation and will warrant further study.

Endogenous human CD19^+^TIM-1^+^ B cells were enriched in the CD73^−^CD71^+^CD25^+^ B cell subset within peripheral blood of healthy human donors, similar to CD19^+^TIM-1^+^ expBreg. Both endogenous CD19^+^TIM-1^+^ B cells and CD73^−^CD71^+^CD25^+^ B cells suppressed autologous CD4^+^ T-cell responses in vitro by CD154-dependent mechanisms, suggesting some homology with the ex vivo*-*expanded human expBreg population.

The development of cancer has often been associated with an over-regulated immune system. We found that the % frequency of the CD73^−^CD71^+^CD25^+^ B cell compartment, as well as TIM-1 and CD154 expression, were enriched in the peripheral blood of patients with SCC when compared to healthy controls. The finding of both TIM-1^+^ B cells and pSTAT3^+^ B cells within B-cell clusters in human SCC tumours may suggest an immunosuppressive microenvironment to promote cancer progression, and could offer novel oncological, therapeutic targets.

The results presented here are promising and exciting but should be interpreted with caution. The longer-term stability of expBreg in vivo, antibody production and the suppressive mechanisms downstream of STAT3 signalling within this human population are unknown. Increasingly, the role of TIM-1 appears to be crucial to the function of human IL-10^+^ Breg. Further work is required to fully elucidate the properties of this dynamic protein as well as to identify its corresponding ligand in this setting. Nonetheless, the ex vivo generation of human CD19^+^TIM-1^+^ Breg with in vivo function described here may pave the way for the development of a human Breg cellular therapy for application in transplantation, autoimmune or inflammatory conditions. Moreover, targeting signalling pathways within human CD19^+^TIM-1^+^ Breg may offer new opportunities for reactivating the immune response in cancer patients.

## Methods

### Mice

Balb/c Rag2^−/−^ cγ^−/−^ mice were obtained from Charles River Laboratories, stock number 14593, and were housed under specific pathogen-free conditions in the Biomedical Services Unit of the John Radcliffe Hospital (Oxford, UK). Mice were housed in individually ventilated cages and handled with gloves. Experimental and control mice were co-housed. Female mice were used for all experiments. Animals received terminal doses of anaesthetic for euthanasia and tissue procurement. All experiments were performed using protocols approved by the Committee on Animal Care and Ethical Review at the University of Oxford and in accordance with the UK Animals (Scientific Procedures) Act 1986, under project license PPL P8869535A and personal license PIL 30/9202, permission granted by the UK Home Office. At the time of the first experimental procedure, mice were between the ages of 6 and 12 weeks. Adoptive transfer of human cells and graft survival experiments using human skin were performed as we have previously described^48,57^. Tissue was harvested and analysed at the rejection endpoint, as previously described^48^. *n* = 6 mice per group were used per experiment, with 3 groups per experiment. Three independent experiments were conducted. Each experiment used a different HLA-mismatched human donor pair. Mice injected with human PBMC were defined as fully reconstituted when human leucocyte chimerism levels of >1% in the spleen or 0.1% in the peripheral blood were established in the absence of GvHD.

### Procurement of human skin

Split-thickness human skin at between 8 and 10/1000 inches thick were harvested with the use of an air-driven dermatome from the anterior abdominal wall of live donors undergoing plastic surgery procedures in the Department of Plastic and Reconstructive Surgery, John Radcliffe Hospital, Oxford. Exclusion criteria included donors who were taking immunosuppressive medication, donors with a primary inflammatory condition or donors with any current or past history of malignancy. Donor age ranged from 28 to 53 years (median 43 years) and donors were all male. Skin was procured with the assistance of Mr Fadi Issa and other members of the Plastic Surgery team. Tissue was stored in RPMI-1640 on ice and was transplanted within 12 h of procurement. Unused skin was destroyed. Tissue for use in animal experiments was obtained with informed patient consent and ethical approval from the Oxfordshire Research Ethics Committee (REC B), study number 07/H0605/130. Blood samples were obtained with informed consent from skin donors for HLA-typing.

### Skin grafting

Skin grafting was performed with full sterile precautions. Mice were placed prone and a 1 × 1 cm piece of skin removed from the left dorsal thorax over the costal margin. A 1 × 1 cm piece of human skin was then fashioned and its edges sutured to the mouse recipient skin with a non-absorbable 8–0 Prolene suture (Ethicon, UK). Grafts were fenestrated and covered with povidone-iodine mesh and pressure dressings which were secured with circumferential tape. Bandages were left in place for 7–10 days and then removed under general anaesthetic. Skin grafts were monitored every 1–2 days until complete loss. Graft assessment was performed independently by two researchers who were both blinded to experimental group allocations.

### Human donors

Blood samples from patients with cutaneous SCC and from healthy human donors, were obtained with informed consent and ethical approval from the NHS Research Ethics Committee, study numbers 12/WS/0288 and 14/SC/0091. For experiments which included samples from SCC patients, blood samples were taken at time-points ranging from 0 to 755 days after the SCC diagnosis had been made (mean time-point of sample was 392 days). SCC patients were age-matched to healthy controls (SCC patients: mean age 81 yrs, age range 72–90 yrs. Healthy controls: mean age 77 yrs, age range 68–86 yrs). All SCC patients and healthy controls were male. When recruiting healthy controls for comparison to SCC patients, participants who had been diagnosed with an autoimmune or inflammatory condition, or a malignancy, were excluded. Formalin-fixed paraffin embedded tumour tissue from 5 cases of human cutaneous SCC were provided by the Oxford Centre for Histopathology Research and the Oxford Radcliffe Biobank. Blood samples from healthy human donors for all other experiments were provided by NHS Blood & Transplant; demographic data was not available for these donors.

### Human CD19^+^ B-cell isolation and generation of expBreg

Human PBMC were isolated from leucocyte cones provided by NHS Blood & Transplant (NHSBT) by Ficoll-Paque (GE Healthcare) gradient centrifugation. Human CD19^+^ B cells were subsequently isolated from PBMC by negative selection using an EasySep^®^ Human B-Cell Enrichment Kit (Stem Cell Technologies), in accordance with manufacturer’s guidelines. Purified human B cells were >96% CD19^+^ as determined by flow cytometry. For all human CD19^+^ B-cell expansion experiments, CD19^+^ B cells were cultured in ‘complete media’ (c.RPMI): RPMI-1640 (Sigma-Aldrich, St. Louis, USA) supplemented with 100 U/μg/ml penicillin/streptomycin (Life Technologies, Carlsbad, USA), L-glutamine and 10% FCS (Gibco, UK). 0.06 × 10^6^ Human CD19^+^ B cells per well were seeded in 96-well U-bottom plates for 7 days. Irradiated CHO cells were co-cultured with human CD19^+^ B cells at ratios of 1:1, 1:5 or 1:10 CHO cells to CD19^+^ B cells in a total volume of 200 µl per well. Unless otherwise stated, CD19^+^ B-cell and irradiated CHO cells were co-cultured with a cytokine combination of 50 U/ml of IL-2, 100 U/ml of IL-4 and 25 U/ml of IL-10 (all PeproTech, Rocky Hill, NJ). In some experiments, human CD19^+^ B cells were co-cultured with agonistic CD40 mAb at 1 μg/ml, 5 μg/ml or 10 μg/ml (R&D Systems, clone 82111) instead of irradiated CHO cells, for 7 days with cytokines IL-2, IL-4 and IL-10. Agonistic CD40 mAb was replaced on day-3 and day-5 of co-culture.

Cultures were fed by replacing culture medium and cytokines every 2–3 days, while irradiated CHO-CD154 cells were renewed on day-3 ± day-7 of expansion. Ratios of CHO-CD154 cells to CD19 were kept constant throughout the culture period. Viable CD19^+^ B cells were harvested on day-7 or day-14 of co-culture, stained with appropriate antibodies (CD19 and PerCP-conjugated 7-AAD viability dye, eBioscience) and sorted to obtain purities of >97% using a BD FacsARIA cell sorter (BD). Both Trypan-Blue exclusion and Calibrite counting beads (BD Biosciences) were used to determine absolute numbers of viable expBreg cells at time-points analysed.

### Culture of Chinese Hamster Ovary (CHO) cells

CD154^+^ and CD154^−^ CHO cell lines were kindly donated by Prof. Claudia Mauri, University College London. All cell lines tested negative for mycoplasma. CHO cells were cultured in DMEM containing 4500 μg/L glucose, 110 μg/L sodium pyruvate and L-glutamine (Sigma-Aldrich) with 100 U/ml penicillin and streptomycin (Life Technologies, Carlsbad, USA) and 5% foetal calf serum (FCS) (Gibco, UK). All cell lines used were routinely checked for mycoplasma contamination. CD154 expression was routinely checked by flow cytometry analysis after staining with CD154-PE mAb (expression >98%). All CHO cell lines were cultured in Corning^®^ T75 flasks and were passaged using standard operating procedure with Trypsin from porcine pancreas (2.5 g/L, Sigma, UK). Before being used in expBreg cell expansion cultures, CHO cells were γ-ray irradiated (65 Gy, 6500 rad) in 50 ml corning tubes for 30 min using a Caesium 137 irradiator.

### PANC-1 cells

PANC-1 cell line was purchased from the American Type Culture Collection (ATCC), catalogue number 1469. All cell lines tested negative for mycoplasma. PANC-1 cells were cultured in DMEM containing 100 U/ml penicillin and streptomycin (Life Technologies, Carlsbad, USA) and 10% foetal calf serum (FCS) (Gibco, UK).

### In vitro suppression assay

To assess the effect of expBreg or Treg cells on proliferation of CD4^+^ T cells, 0.05 × 10^6^ expBreg or Treg cells were co-cultured with autologous CD4^+^ T cells or PBMC for 5 days in 96-well U-bottom plates in a total volume of 200 μl per well. CD4^+^ T cells and PBMC (responder cells) were stained with either CFSE or VPD. Dye-stained responder cells were stimulated with anti-CD3/CD28 beads at a ratio of 1:10 beads to responder cells. Stimulated CD4^+^ T cells or PBMC cultured in the absence of expBreg, or instead with nCD19^+^ B cells, served as control conditions. On day 5 of culture, cells were harvested, stained for cell-surface markers and analysed by flow cytometry. In some experiments, IL-21 [0.05 μg/ml] or hIL-2 [10, 100, or 1000 μg/ml] were added to suppression assays at day 0. % Inhibition of proliferation is an expression of Division Index (DI) of live CD4^+^CFSE^+^ T cells at day 5 relative to that of the stimulated CD4^+^ T-cell control 0:1, such that % Inhibition of proliferation = (1 − (DI of CD4^+^ T cells_experimental condition_/DI of stimulated CD4^+^ T cells_control_)) × 100.

To assess the effect of expBreg on intracellular cytokine production by CD4^+^ T cells, expBreg cells were co-cultured with CD4^+^ T cells and anti-CD3/CD28 beads for 72 h. 2% PMA, 1% ionomycin and 1% brefeldin (PIB) were added to cultures for the last 6 h of culture; cells were then harvested, surface-stained, permeabilised and stained intracellularly for Th1 cytokines interferon-γ (IFNγ) and tumour necrosis factor α (TNFα). In some experiments, non-expanded CD19^+^ B cells were co-cultured with CD4^+^ T cells and plate-bound anti-CD3 mAb [0.5 μg/ml] for 72 h, and PIB added for the last 6 h of culture before intracellular staining for TNFα expression.

### Mechanistic in vitro blockade

To determine mechanisms by which expBreg cells suppressed proliferation or cytokine production by autologous CD4^+^ T cells, different inhibitory reagents were added to in vitro suppression co-cultures on day 0 of the assay, unless otherwise stated. Inhibitory agents were added to both control and experimental conditions and expBreg-mediated suppression calculated relative to the control condition which had also been exposed to the inhibitory reagent. Inhibitory concentrations were determined by titration in excess of the ND_50_ provided by the manufacturer. When necessary, appropriate isotype mAbs were also used in control conditions. In some experiments, blocking monoclonal antibodies were added to co-cultures of CD19^+^ B cells and CD154^+^ CHO cells for the last 48 h of 7-day expansion cultures, harvested and then washed x3 prior to addition to suppression assays and/or undergoing pSTAT3 staining. In some experiments, blocking monoclonal antibodies were added to expBreg immediately after harvest, for 1 h, before washing x3 prior to addition to suppression assays and/or undergoing pSTAT3/5 staining. In some experiments, expBreg were pre-incubated with a STAT3 inhibitor or DMSO ± hIL-21 [0.05 μg/ml] for 2 h and washed x3 prior to addition to suppression assays.

Full details of antibodies and concentrations used for mechanistic studies are provided in Supplementary Table 1. Reagents purchased from R&D Systems include: TGFβ inhibitor SB 431542 (4-[4-(1,3-benzodioxol-5-yl)-5-(2-pyridinyl)-1*H*-imidazol-2-yl]benzamide), [10 μM/L]; anti-CD154 mAb, clone 40804 [10 μg/ml]; anti-CD40 mAb, clone 82102 [10 μg/ml]; anti-IL-10 mAb, clone 23738 [10 μg/ml]; anti-IL-10Rα mAb, clone 37607 [10 μg/ml]; anti-CD122 mAb, clone 27302 [10 μg/ml]; anti-CD25 mAb, clone 22722 [10 μg/ml]; anti-CD25 mAb, clone 24212 [10 μg/ml]; anti-CD86 mAb, clone 37301 [10 μg/ml]; CD80 mAb, clone 37711 [10 μg/ml]; FAS-L mAb, clone 100419 [10 μg/ml]; PD-1 mAb [10 μg/ml], cat no: AF1086; IL-6 mAb, clone 1936 [10 μg/ml]; IL-6R mAb, clone 17506 [10 μg/ml]; IgG_1κ_ mAb [10 μg/ml]; IgG_2aκ_ mAb [10 μg/ml]. Reagent purchased from Biolegend include: anti-TIM-1 mAb, clone 1D12 [10 μg/ml]. Reagent purchased from Santa Cruz Biotech: Stat3 Inhibitor III WP 1066 [10 μM]. In some mAb-blockade experiments, expBreg which had been pre-incubated with blocking mAbs and then washed, were subsequently stained with fluorescence-conjugated mAbs of the same clones and assessed by flow cytometry to assess binding of the blocking mAb. For TIM-blockade: anti-TIM-1 mAb (Biolegend), clone 1D12 [10 μg/ml] was used for blocking; PE-conjugated TIM-1 mAb (Biolegend), clone 1D12 was used for flow cytometry. For CD154-blockade: anti-CD154 mAb (R&D Systems), clone 40804 [10 μg/ml] was used for blocking; APC-conjugated CD154 mAb (R&D Systems), clone 40804 was used for flow cytometry. For CD25-blockade: anti-CD25 mAb (R&D Systems), clone 24212 [10 μg/ml] was used for blocking; PE-conjugated CD25 mAb (R&D Systems), clone 24212 was used for flow cytometry.

### Flow cytometry

Samples were acquired and sorted using a FACSAria (BD Biosciences, UK) whilst flow cytometric data were acquired using BD Canto II (BD Biosciences, UK), and analysed using FlowJo software v10.7.1 (Flowjo Enterprise, USA).

Full details of antibodies and concentrations used for staining are provided in Supplementary Table 1. Anti-human monoclonal antibodies purchased from BD Pharmingen include: APC-Cy7-conjugated CD19 (SJ25C1), PE-Cy7-conjugated CD25 (M-A251), PE-conjugated CD24 (ML5), FITC-conjugated CD38 (HIT2), PE-conjugated CD127 (H1L-7R-M21), Alexa Fluor 647-conjugated pSTAT3 (pY705). Anti-human monoclonal antibodies purchased from eBioscience include: PE-Cy7-conjugated CD20 (2H7), PE-Texas Red-conjugated CD4 (RPA-T4), FITC-conjugated CD8 (RPA-T8), PE-conjugated γCR (TUGh4), eFluor 450-conjugated CD27 (O323), eFluor 450-conjugated CD138 (HB7), APC-conjugated CD1d (51.1), FITC-conjugated CD5 (L17F12), eFluor 450-conjugated CD21 (HB5), APC-conjugated CD71 (OKT9), eFluor 450-conjugated CD73 (AD2), PE-conjugated LAP (FNLAP), eFluor 660-conjugated IL-10 (JES3-9D7), FITC-conjugated TNFα (MAb11), PE-conjugated IFNγ (4S.B3), PE-conjugated CD154 (24-31), APC-conjugated CD40 (5C3). Anti-human monoclonal antibodies purchased from Biolegend include: PE-conjugated TIM-1 (1D12), APC-conjugated IL-10R (3F9), APC-conjugated CD122 (TU27). Anti-human monoclonal antibody purchased from Beckman Coulter include: PE-Texas Red-conjugated CD10 (ALB-1). Anti-human monoclonal antibody purchased from Invitrogen: APC-conjugated CD45 (HI30). Anti-human monoclonal antibodies purchased from R&D Systems include: APC-conjugated CD154 mAb (40804), PE-conjugated CD25 mAb (24212).

#### Cell-surface marker staining

Cells were first incubated with human sera for 15 min at 4 °C to reduce non-specific antibody binding. Cells were then incubated with the appropriate fluorochrome-coupled mAbs for 45 min at 4 °C in the dark. Cells were then washed with PBS and spun for 5 min at 1500 rpm at 4 °C before flow cytometry analysis. Fluorescence-minus-one (FMO) controls were used as standard controls to permit accurate gating of positive and negative populations.

#### Intracellular staining

After appropriate cell-surface staining, cells were permeabilised using a BD Cytofix/Cytoperm Kit (BD Biosciences, UK) in accordance with manufacturer’s guidelines. Cells were then incubated with either the fluorochrome-coupled mAb under interrogation or the appropriate isotype control for 1 h at 4 °C in the dark. In some experiments, fluorescence-minus-one controls (FMO) were used. Cells were washed with PBS and spun for 5 min at 1500 rpm at 4 °C before flow cytometry analysis. In order to measure expression of IL-10, cells were first incubated for 6 h with PMA, ionomycin and monensin (PIM) in c.RPMI before staining.

#### pSTAT3 and pSTAT5 staining

expBreg cells were harvested from expansion co-cultures, initially rested in c.RPMI for 90 min and then washed to allow any existing pSTAT3 or pSTAT5 to degrade. 0.1 × 10^6^ expBreg cells were subsequently incubated in 100 μl c.RPMI for 10 min at 37 °C, with exogenous hIL-10 [0.01 μg/ml], hIL-21 [0.05 μg/ml], hIL-2 [10^4^ U/ml] or hIL-4 [1 μg/ml] (or c.RPMI as a control) in 96-well, v-bottom plates. 100 μl/well of Cytofix fixation Buffer (BD Biosciences, UK) was added to cell culture for an additional 10 min incubation period at 37 °C. Cells were subsequently spun for 5 min at 1500 rpm at 4 °C and supernatants decanted. 100 μl/well of Phosflow Perm Buffer (BD Biosciences, UK) was added to cell culture and cells were incubated for 30 min on ice. Cells were then washed twice with PBS at 1500 rpm for 5 min at 4 °C before staining with Alexa Fluor 647-conjugated pSTAT3 antibody (BD Pharmingen) or Alexa Fluor 488-conjugated pSTAT5 (pY694) antibody (BD Biosciences) for 1 h at 4 °C. Cells were washed with PBS at 1500 rpm for 5 min at 4 °C before immediate FACS analysis. FMO controls were used to assist accurate gating. In some experiments, expBreg underwent IL-10 exposure and pSTAT3 staining without an initial 90 min rest period. CD4^+^ T cells, nCD19^+^ B cells and unstained expBreg cells were included as relevant controls.

### Gene editing with CRISPR-Cas9

For experiments using chemically modified sgRNAs, guides were ordered from Synthego Biosciences with 2′-O-methyl and 3′ phosphorothioate internucleotide modified linkages and Synthego-modified EZ Scaffolds at the 3′ and 5′ ends of sgRNAs. Gene Knockout Kits v2 (Synthego) which included multiple sgRNAs (mgRNA) for each protein target, were utilised in accordance with manufacturer’s guidelines. mgRNA was initially reconstituted with TE Buffer (Synthego) to achieve [100 μM] concentration. Cas9 nuclease was purchased from Synthego (300pmole at 20 μM). sgRNA sequences which made up the mgRNA pool for TIM-1 (HAVCR1) were: UCCAGACAAUGCCAUUU; CAGGGUAGUGUGACAGA; UUUGCAGAUUCUGUAGC. sgRNA sequences which made up the mgRNA pool for CD154 (CD40LG) were: CAAAAUAGAUAGAAGAUGAA; ACGAUACAGAGAUGCAACAC; CCAGUUUGAAGGCUUUGUGA. To generate ribonucleoproteins (RNPs), mgRNA at [100μM] was initially diluted with Nuclease-free water (Synthego) to obtain [30 μM] working stock. mgRNA was subsequently complexed with Cas9 protein and TE Buffer at final concentration of [6 μM] mgRNA and [6 μM] Cas9 protein, for 20 min at room temperature to generate RNPs. Each RNP was formulated at a total volume of 10 μl per condition. Each condition was set up in triplicate per human donor. Additional control conditions when generating RNPs included: mgRNA + TE Buffer; Cas9 + TE Buffer; TE Buffer only. CRISPR efficiency was assessed by flow cytometry analysis of target protein expression.

### Electroporation

For electroporations, human B cells were initially reconstituted at 10 × 10^6^/ml concentration in Buffer T (Thermo Fisher Scientific). In experiments where expBreg were electroporated, expBreg were reconstituted in Buffer T after harvesting and washing, following 7-day expansion with 50 U/ml of IL-2, 100 U/ml of IL-4 and 25 U/ml of IL-10 as described earlier in the Methods section. 100 μl of 10 × 10^6^/ml B cells (i.e. 1 × 10^6^ B cells) were added per well per electroporation, to a flat-bottomed 96-well plate. 10 μl of RNP were added to each well of 100 μl of 1 × 10^6^ human B cells. Each RNP condition (mgRNA + Cas9 + TE Buffer; mgRNA + TE Buffer; Cas9 + TE Buffer; TE Buffer only) was set up in triplicate per human donor. Human B cells were now ready for electroporation in the Neon Transfection System (Thermo Fisher Scientific). Cells were loaded in 100 μl tips and electroporated in accordance with the manufacturer’s instructions using settings of 1400 V, 10 ms width, and 3 pulses^38^. Electroporated B cells were immediately co-cultured with irradiated CD154^+^ CHO cells at 1:10 ratio of CHO cells:B cells and cytokines IL-2 (50 U/ml), IL-4 (100 U/ml) and IL-10 (25 U/ml) in a total volume of 1 ml per well of a flat-bottomed 24 well plate, for 72 h in an incubator at 37 °C. The irradiated CD154^+^ CHO cells and cytokines had been pre-warmed in an incubator at 37 °C for 1 h prior to the addition of electroporated B cells. B cells were subsequently harvested, washed, and stained for flow cytometry analysis± added to suppression assays.

### ELISA

Levels of human cytokines IL-35 and IL-10 in in vitro culture supernatants were measured using a Human IL-35 Heterodimer ELISA Kit (Biolegend) and a Human IL-10 ELISA kit (BD Biosciences) respectively, in accordance with manufacturers’ guidelines.

### Cytokine bead array

Levels of human cytokines IL-2, IL-10, IL-4, IL-6 and IL-17a as well as human immunoglobulin IgG (total) and IgM in humanised Balb/c Rag2^−^^/−^ cγ^−^^/−^ mouse sera or in supernatants taken from in vitro cultures were measured using a Human Cytokine Bead Array Enhanced Sensitivity Flex Set system (BD Biosciences), in accordance with manufacturer’s guidelines. All data was processed using FCAP v1.0 analysis software (BD Biosciences).

### Immunohistochemistry and image analysis

Five cases of primary cutaneous invasive SCC were identified through review of diagnostic hematoxylin and eosin (H&E) stained slides. Strictly serial 4 μm sections were cut from the corresponding formalin-fixed paraffin embedded tissue blocks. These slides underwent double immunohistochemistry staining on a Leica BOND-MAX automated staining machine (Leica Biosystems); human tonsil sections acted as a positive control specimen for each staining run. Briefly, sections were deparaffinized, underwent epitope retrieval and endogenous peroxidase activity was blocked with 3% hydrogen peroxide (5 min). Subsequently, sections were incubated with the first primary antibody (30 min) followed by post-primary and polymer reagents (8 min each). Next, 3,3′-Diaminobenzidine (DAB) chromogen was applied (10 min) (all reagents contained within the BOND Polymer Refine Detection kit, Leica Biosystems, catalogue no. DS9800). The sections then underwent a second epitope retrieval step before incubation with the second primary antibody (30 min) followed by post-primary and polymer reagents (8 min each). Next, Fast red chromogen was applied (15 min) and the sections were counterstained with hematoxylin (5 min) (all reagents contained within the BOND Polymer Refine Red Detection kit, Leica Biosystems, catalogue no. DS9390). Slides were mounted with a glass coverslip and left to dry overnight. The following primary antibodies were used during staining, at concentrations recommended by their manufacturers: CD20 (Leica Biosystems, L26, 1:100), pSTAT3 (Abcam, ab76315, 1:100), TIM-1 (Abcam, ab47635, 1:100) and PAX5 (Abcam, ab109443, 1:500).

Diagnostic H&E slides were scanned at x400 magnification using the NanoZoomer S210 digital slide scanner (Hamamatsu). Double-stained immunohistochemistry slides were scanned at ×400 magnification on a Vectra^®^ Polaris™ Automated Quantitative Pathology Imaging System (PerkinElmer), which permits brightfield spectral un-mixing to achieve colour deconvolution of DAB, Fast red and hematoxylin stains (spectra of these dyes were defined in advance of unmixing by analysing single stained human tonsil sections). Digital images were reviewed and manually annotated by a trained pathologist (PSM) and figures were created in Photoshop (Adobe).

### Tissue typing

5 ml of whole blood was obtained from skin donors and leucocyte cones. These samples were analysed by the Oxford Transplant Centre Histocompatibility and Genetics Laboratory. Full tissue typing was performed for HLA-A, -B, -Cw, -DR and –DQ.

### Statistical analysis

Log-rank tests, non-parametric Mann–Whitney *U* tests and parametric Student *t* tests were applied as appropriate and as detailed in figure legends. For multiple comparisons of parametric and paired data, One-way ANOVA with Tukey’s multiple comparisons test was used. For multiple comparisons of non-parametric and unpaired data, One-way ANOVA with Dunn’s multiple comparisons test was used. Statistical analysis was performed using Graphpad Prism v9.0 software. *p* values <0.05 were considered significant.

### Reporting summary

Further information on research design is available in the Nature Research Reporting Summary linked to this article.

## Supplementary information

Supplementary Information

Reporting Summary

## Data Availability

The authors declare that all relevant data supporting the findings of this study are available within the paper and its Supplementary Information files. Source data are provided with this paper.

## Source data

Source Data
