# Supplementary material for: Ex vivo-expanded human CD19+TIM-1+ regulatory B cells suppress immune responses in vivo and are dependent upon the TIM-1/STAT3 axis
Source: Nat Commun. 2022 Jun 3;13:3121. doi: 10.1038/s41467-022-30613-z (PMC9166804; doi:10.1038/s41467-022-30613-z)
Supplement: Supplementary file 1 — Supplementary Information [file 41467_2022_30613_MOESM1_ESM.pdf]

Supplementary Information

Ex vivo-expanded human $CD19^{+}TIM\text{-}1^{+}$ regulatory B cells suppress immune responses *in vivo* and are dependent upon the TIM-1/STAT3 axis

***Shankar S[1,2], Stolp J[1], Juvet SC[1], Beckett J[1], Macklin PS[3], Issa F[1], Hester J[1], *Wood KJ[1]**

Affiliations

1 Transplantation Research and Immunology Group, Nuffield Department of Surgical Sciences, University of Oxford, Oxford UK

2 Oxford Transplant Centre, Nuffield Department of Surgical Sciences, University of Oxford, Oxford UK

3 Nuffield Department of Medicine Research Building, Nuffield Department of Medicine, University of Oxford, Oxford, United Kingdom

*Correspondence to: Sushma Shankar sushma.shankar@nds.ox.ac.uk and Kathryn J Wood kathryn.wood@nds.ox.ac.uk

Table of Contents

Supplementary Figure 1 3

Supplementary Figure 2 5

Supplementary Figure 3 7

Supplementary Figure 4 9

Supplementary Figure 5 11

Supplementary Figure 6 13

Supplementary Figure 7 15

Supplementary Figure 8 17

Supplementary Figure 9 19

Supplementary Figure 10 21

Supplementary Table 1 23

Supplementary Figure 1

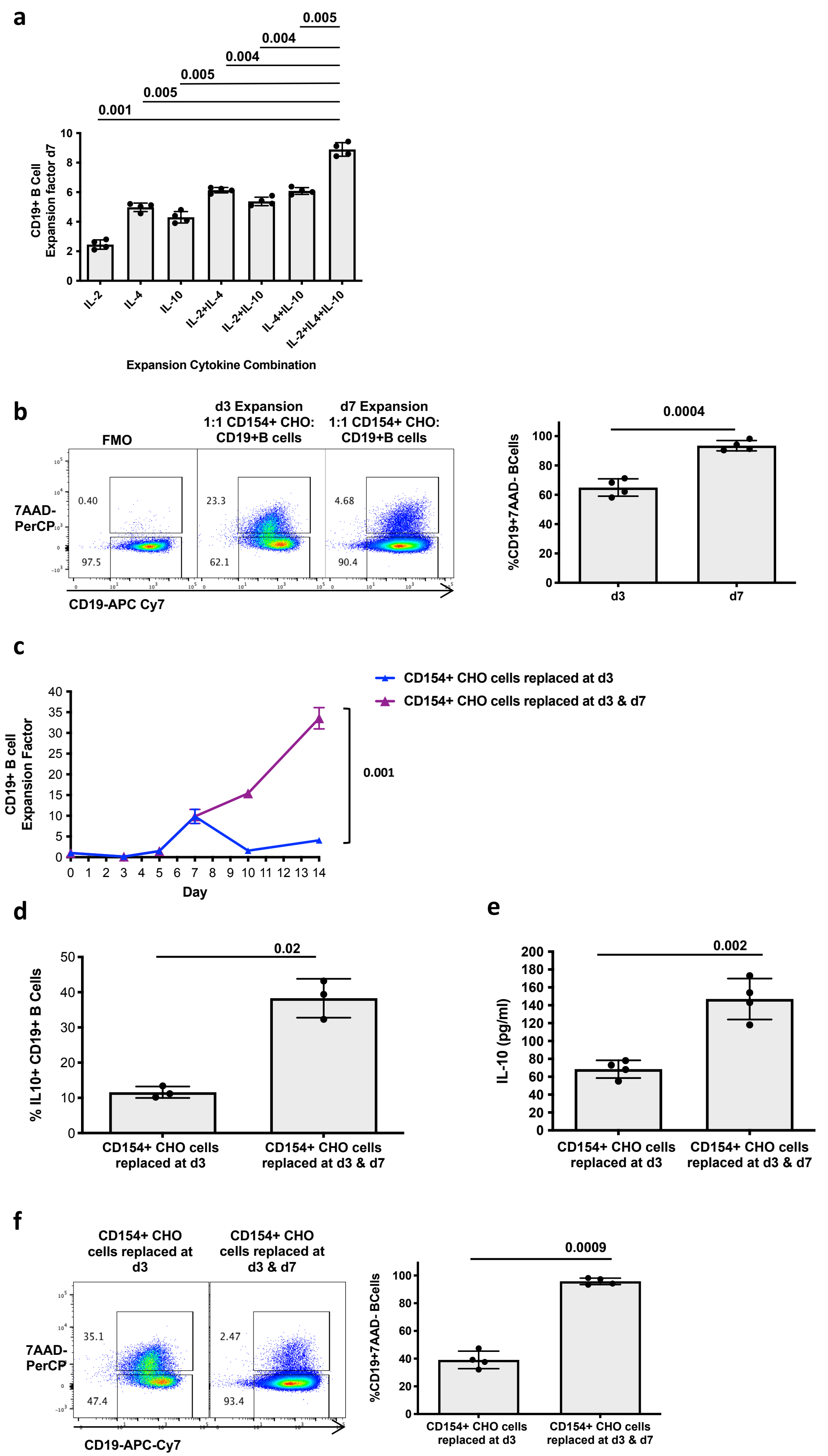

Supplementary Figure 1

a) Summarised data of expansion factor of expBreg when expanded at a 1:1 $CD154^+$ CHO cell-$CD19^+$ B cell ratio with different cytokine combinations are presented. Data from experiments performed with cells from different healthy donors ($n = 4$) are presented. Each dot is an individual response. **(b)** Representative FACS plots and summarised data of % $CD19^+7AAD^+$ B cells at day-3 and day-7 of B cell expansion are shown. Data from experiments performed with cells from different healthy donors ($n = 4$) are presented. Each dot is an individual response. **(c)** Human $CD19^+$ B cell expansion could be maintained in culture for at least 14 days when $CD154^+$ CHO cells were replaced at day-3 and day-7 within the *ex vivo* culture system. Data from experiments performed with cells from different healthy donors ($n = 3$) are presented. **(d)** Summarised data of % $CD19^+IL\text{-}10^+$ B cells at day-14 of B cell expansion are shown, when $CD154^+$ CHO cells were replaced either at day-3 only, or at day-3 and day-7 within the *ex vivo* culture system. Data from experiments performed with cells from different healthy donors ($n = 3$) are presented. Each dot is an individual response. **(e)** Summarised data of IL-10 production by human $CD19^+$ B cells at day-14 of B cell expansion are shown, when $CD154^+$ CHO cells were replaced either at day-3 only, or at day-3 and day-7 within the *ex vivo* culture system. Data from experiments performed with cells from different healthy donors ($n = 4$) are presented. Each dot is an individual response. **(f)** Representative FACS plots and summarised data of % $CD19^+7AAD^+$ B cells at day-14 of B cell expansion are shown, when $CD154^+$ CHO cells were replaced either at day-3 only, or at day-3 and day-7 within the *ex vivo* culture system. Data from experiments performed with cells from different healthy donors ($n = 4$) are presented. Each dot is an individual response. Error bars in each panel represent Mean +/- SD **(a, b, d, e, f)**. One-way ANOVA with Tukey's multiple comparisons test **(a)** and 2-tailed paired t-test **(b, c, d, e, f)** were used. Source data are provided as a Source Data file.

Supplementary Figure 2

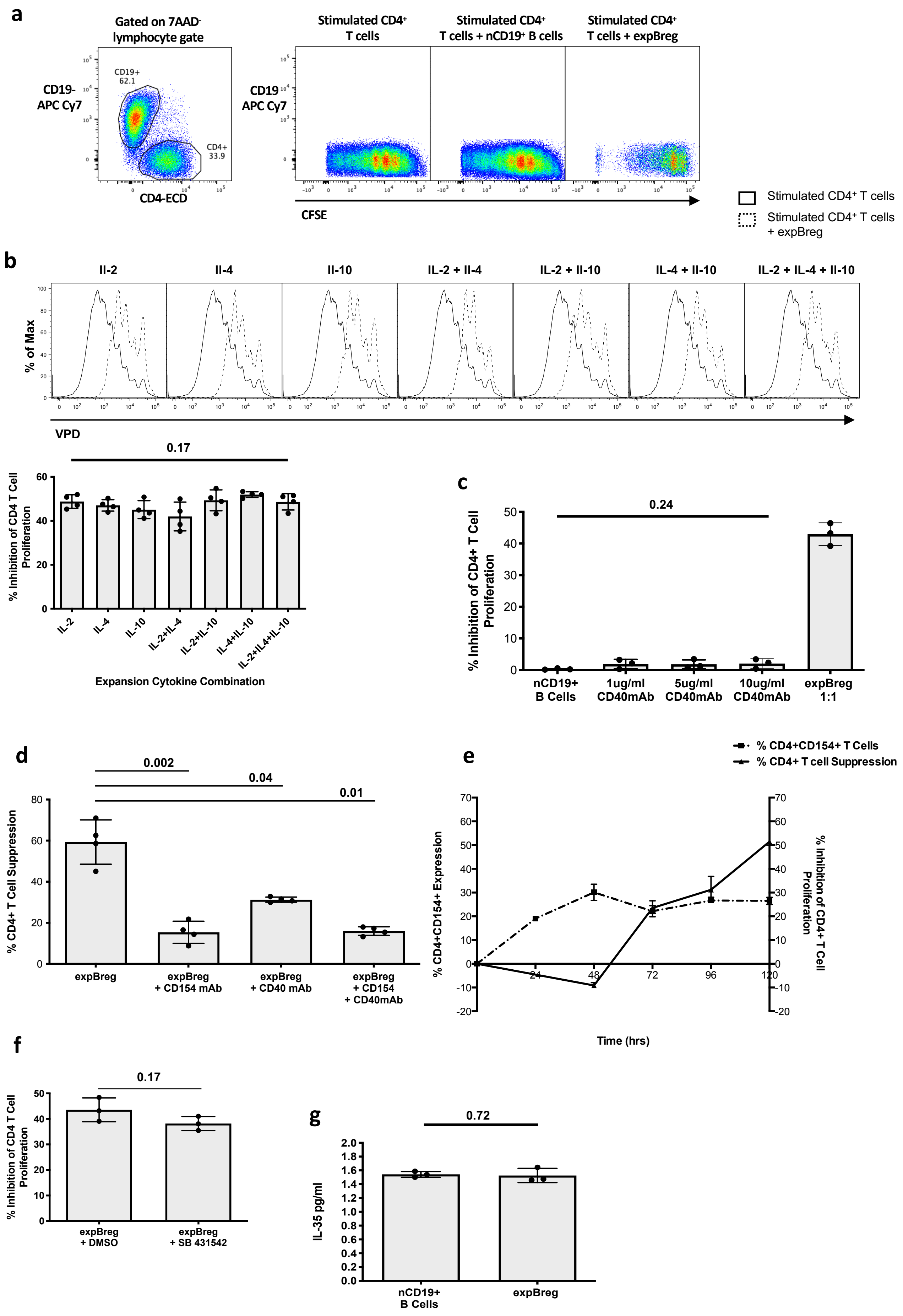

Supplementary Figure 2

(a) Gating strategy & representative FACS plots of live $CD4^+CFSE^+$ T cells when autologous $CD4^+CFSE^+$ T cells were cultured with anti-CD3/CD28 beads for 5 days +/- non-expanded $CD19^+$ B cells or expBreg at 1:1 ratio of B:T cells. Live/dead exclusion was performed using 7-AAD. **(b)** Representative histograms of live $CD4^+VPD^+$ T cells and summarised data of suppressive potency of expBreg when expanded at a 1:1 $CD154^+$ CHO cell-$CD19^+$ B cell ratio with different cytokine combinations, are presented. Autologous $CD4^+VPD^+$ T cells were cultured with anti-CD3/CD28 beads for 5 days +/- expBreg. % Inhibition of $CD4^+$ T cell proliferation is an expression of Division Index of live $CD4^+CFSE^+$ T cells at day 5 relative to that of the Stimulated $CD4^+$ T cell control. Data from experiments performed with cells from different healthy donors ($n = 4$) are presented. Each dot is an individual response. **(c)** Summarised data of % inhibition of CD4+ T cell proliferation are presented, when autologous $CD4^+VPD^+$ T cells were cultured with anti-CD3/CD28 beads for 5 days +/-non-expanded $CD19^+$ B cells (n$CD19^+$ B cells), expBreg or $CD19^+$ B cells that had been stimulated with plate-bound agonistic CD40 mAb (at [1ug/ml], [5ug/ml] or [10ug/ml] concentrations) for 7 days. Data from experiments performed with cells from different healthy donors ($n = 3$) are presented. Each dot is an individual response. **(d)** Summarised data of % inhibition of $CD4^+$ T cell proliferation by expBreg when in the presence of blocking CD154 mAb, blocking CD40 mAb, or both. Blocking mAbs were added at day-0 of the 5-day suppression assay. Data from experiments performed with cells from different healthy donors ($n = 4$) are presented. Each dot is an individual response. **(e)** Summarised data of % Inhibition of $CD4^+$ T cell proliferation by expBreg and expression of CD154 on $CD4^+$ T cells when measured daily in the 5-day suppression assay. Data from experiments performed with cells from different healthy donors ($n = 4$) are presented. Each dot is an individual response. **(f)** Summarised data of % inhibition of $CD4^+$ T cell proliferation by expBreg when in the presence of TGFβ inhibitor SB 431542 [μM/L] or DMSO. SB 431542 or DMSO were added at day-0 of the 5-day suppression assay. Data from experiments performed with cells from different healthy donors ($n = 3$) are presented. Each dot is an individual response. **(g)** Summarised data of IL-35 production by n$CD19^+$ B cells or expBreg. Data from experiments performed with cells from different healthy donors ($n = 3$) are presented. Each dot is an individual response. Error bars in each panel represent Mean +/- SD **(b, c, e, f, g)**. One-way ANOVA with Tukey's multiple comparisons test **(b, c, d)** and 2-tailed paired t-test **(f, g)** were used. Source data are provided as a Source Data file.

Supplementary Figure 3

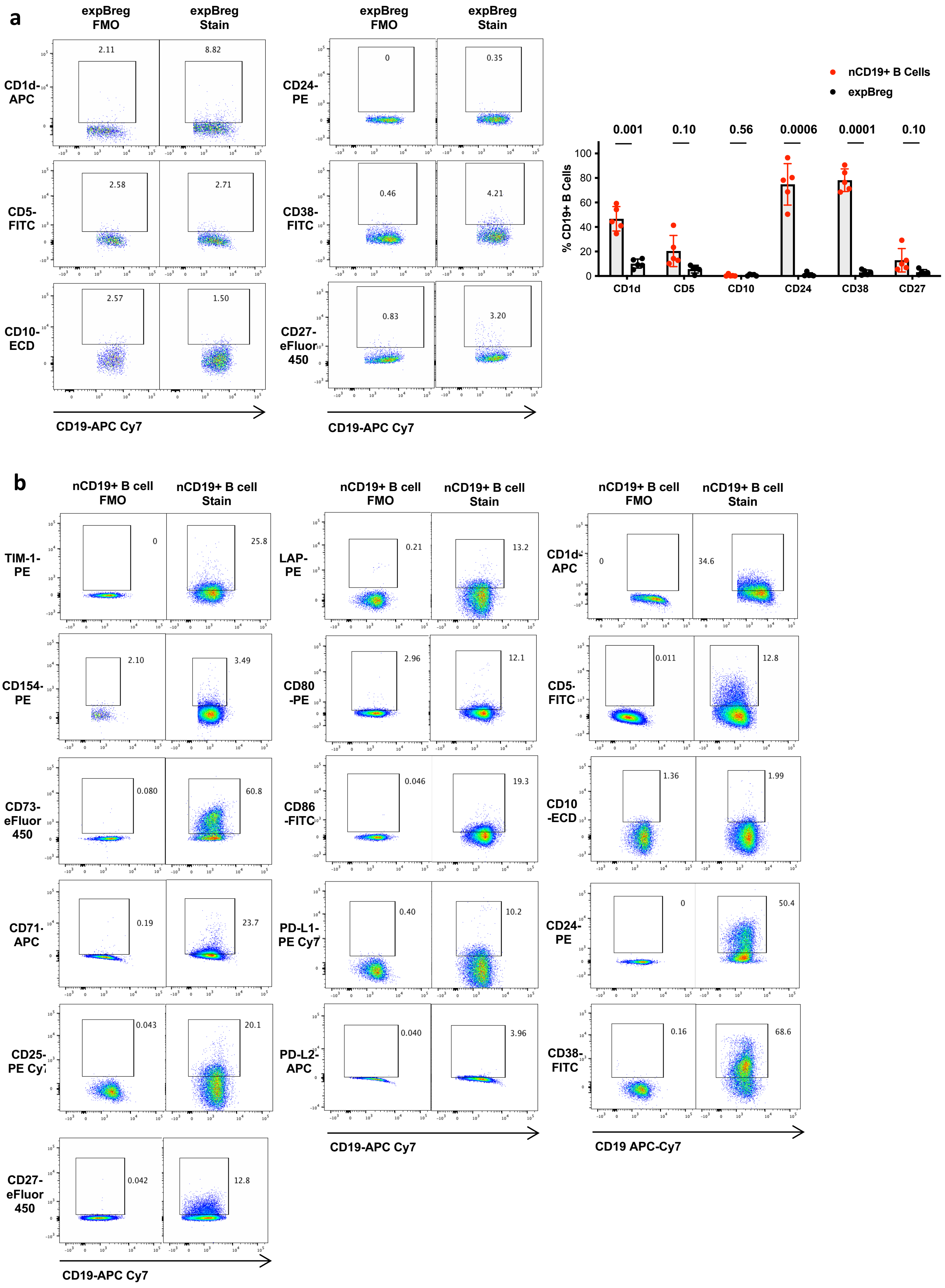

Supplementary Figure 3

(**a**) Representative FACS plots of live $CD19^+$ B cells and summarised data demonstrating expression of cell surface markers by expBreg. Gating is on FMO controls. % Expression is compared between expBreg and autologous non-expanded $CD19^+$ B cells ($nCD19^+$ B cells). Data from experiments performed with cells from different healthy donors ($n = 5$) are presented. Each dot is an individual response. **(b)** Representative FACS plots of live $CD19^+$ B cells demonstrating expression of cell surface markers by non-expanded $CD19^+$ B cells ($nCD19^+$ B cells). Gating is on FMO controls. Data from experiments performed with cells from different healthy donors ($n = 5$) are presented. Each dot is an individual response. Error bars in **(a)** represent Mean +/- SD. 2-tailed paired t-tests **(a)** were used. Source data are provided as a Source Data file.

Supplementary Figure 4

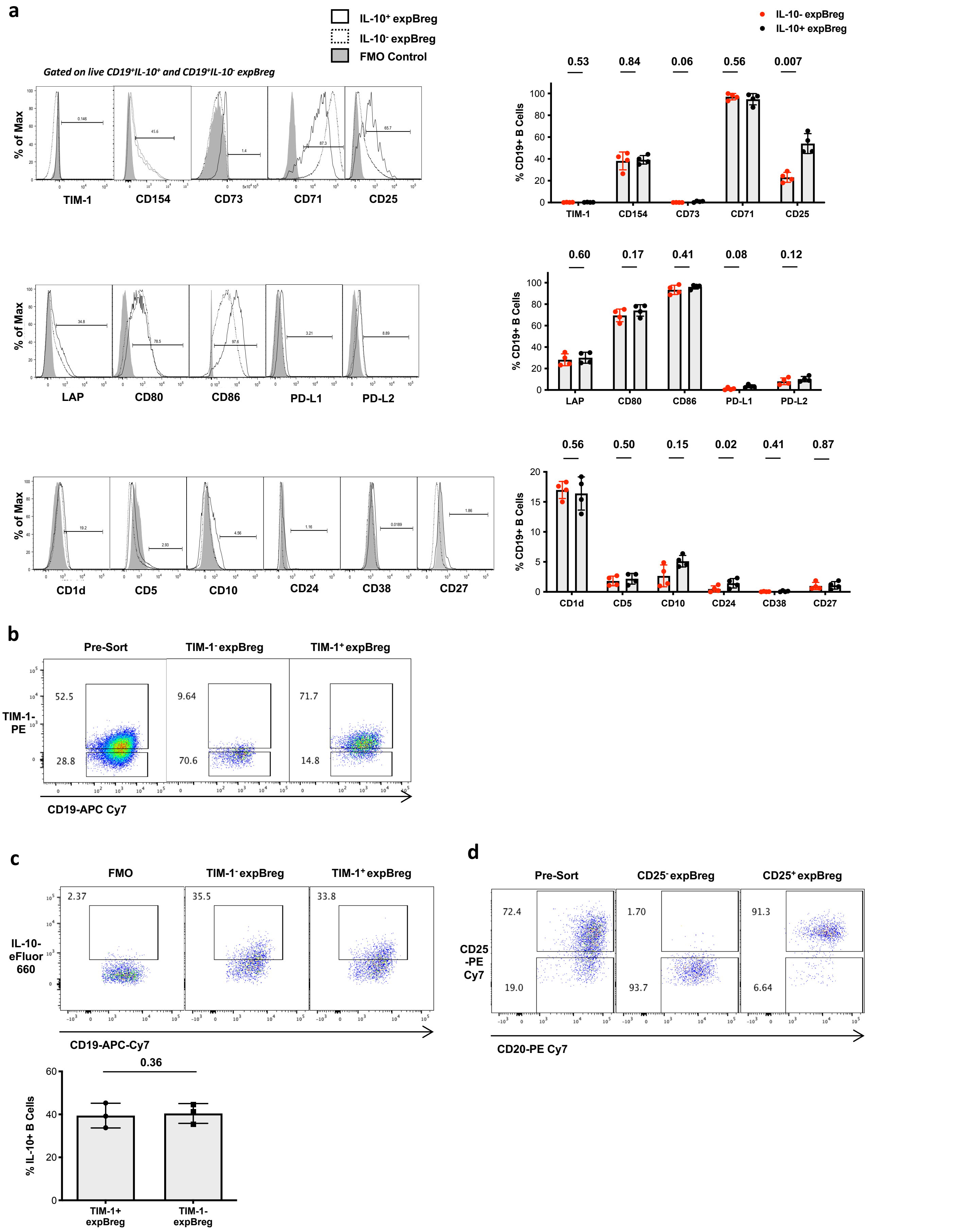

Supplementary Figure 4

(a) Representative histograms and summarised data of cell surface markers expressed by IL-10^{+} and IL-10^{-} CD19^{+} expBreg are presented. Gating is on FMO controls. Data from experiments performed with cells from different healthy donors ($n = 4$) are presented. Each dot is an individual response. **(b)** FACS-sorting strategy to isolate expBreg based on the expression of TIM-1 **(c)** Representative FACS plots and summarised data of IL-10 expression by FACS-sorted TIM-1^{+} and TIM-1^{-} expBreg. Gating is on fluorescence minus one controls (FMO). **(d)** FACS-sorting strategy to isolate expBreg based on the expression of CD25. Error bars in **(a, c)** represent Mean +/- SD. 2-tailed paired t-tests **(a, c)** were used. Source data are provided as a Source Data file.

Supplementary Figure 5

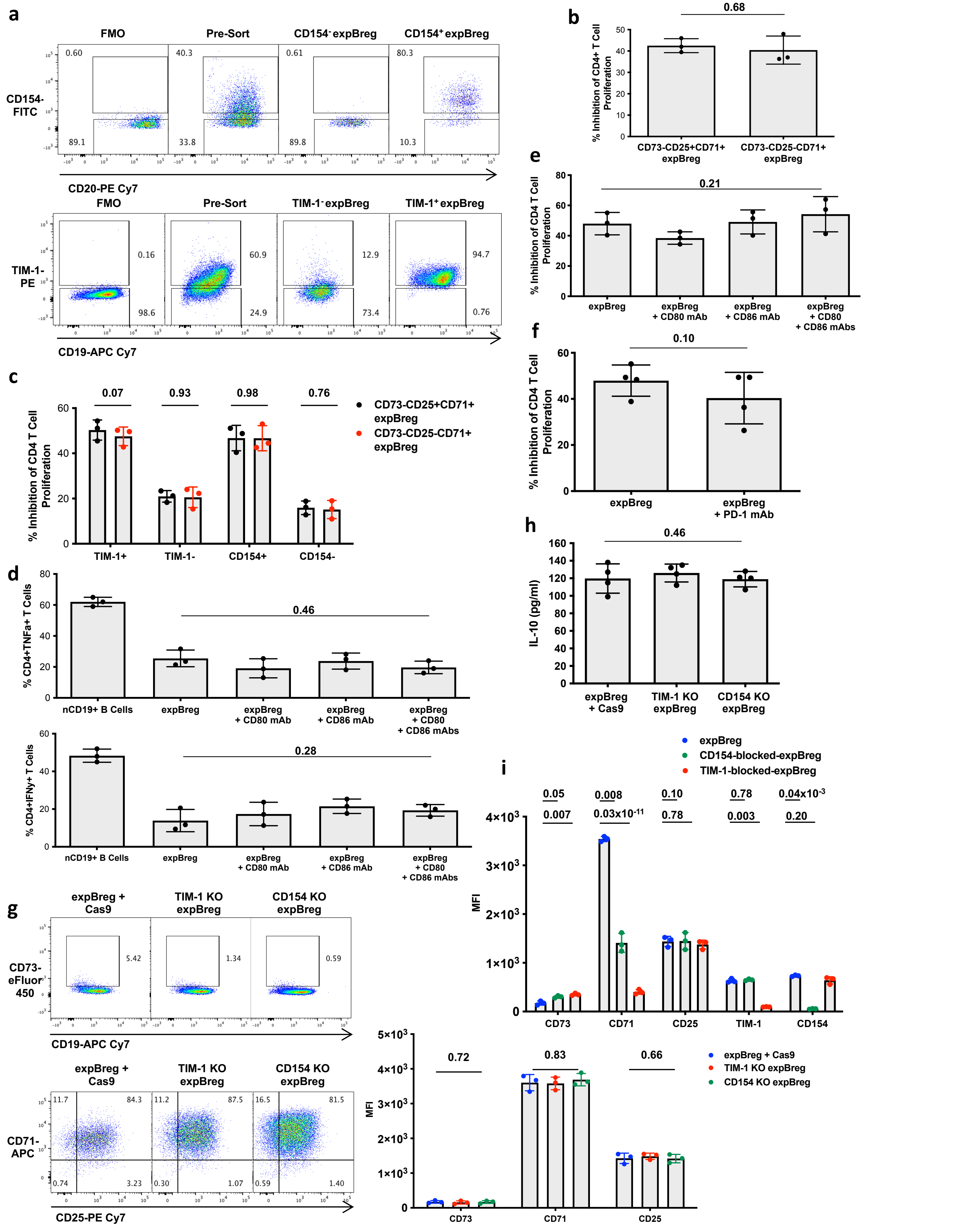

Supplementary Figure 5

(a) FACS-sorting strategy to isolate expBreg based on the expression of CD154 or TIM-1. Gating is on fluorescence minus one controls (FMO). **(b)** Summarised data of % inhibition of $CD4^+$ T cell proliferation by FACS-sorted $CD73^-CD25^+CD71^+$ and $CD73^-CD25^-CD71^+$ expBreg are presented. Data from experiments performed with cells from different healthy donors (*n* = 3) are presented. Each dot is an individual response. **(c)** Summarised data of % inhibition of $CD4^+$ T cell proliferation by FACS-sorted $CD73^-CD25^+CD71^+$ and $CD73^-CD25^-CD71^+$ expBreg which had been further FACS-sorted based on TIM-1 and CD154 expression, are presented. Data from experiments performed with cells from different healthy donors (*n* = 3) are presented. Each dot is an individual response. **(d)** Summarised data of % $CD4^+TNFa^+$ and $CD4^+IFNy^+$ T cells at day-3 of suppression assay when autologous $CD4^+$ T cells were cultured with anti-CD3/CD28 beads +/- expBreg when in the presence of blocking CD80 mAb, blocking CD86 mAb, or both. Blocking mAbs was added at day-0 of suppression assay. Data from experiments performed with cells from different healthy donors (*n* = 3) are presented. Each dot is an individual response. **(e)** Summarised data of % inhibition of $CD4^+$ T cell proliferation by expBreg when in the presence of blocking CD80 mAb, blocking CD86 mAb, or both. Blocking mAbs were added at day-0 of suppression assay. Data from experiments performed with cells from different healthy donors (*n* = 3) are presented. Each dot is an individual response. **(f)** Summarised data of % inhibition of $CD4^+$ T cell proliferation by expBreg when in the presence of blocking PD-1 mAb. Blocking mAb was added at day-0 of suppression assay. Data from experiments performed with cells from different healthy donors (*n* = 4) are presented. Each dot is an individual response. **(g)** Representative FACS plots of live $CD19^+$ expBreg and summarised data are presented which demonstrate protein expression of CD73, CD25 and CD71 after electroporation with Cas9 alone +/- TIM-1 or CD154 multiguide RNAs to generate TIM-1 or CD154 knockouts (KO). **(h)** Summarised data of IL-10 production by Cas9-expBreg, TIM-1 KO or CD154 KO expBreg are presented. Data from experiments performed with cells from different healthy donor (*n* = 4) are presented. Each dot is an individual response. **(i)** Summarised data of mean fluorescence intensity (MFI) of cell surface markers expressed by expBreg which had been pre-incubated with IgG isotype control (Isotype-expBreg), anti-CD154 mAb (CD154-blocked-expBreg) or anti-TIM-1 mAb (TIM-1-blocked-expBreg) for the last 48 hours of expansion, are presented. Data from experiments performed with cells from different healthy donors (*n* = 3) are presented. Each dot is an individual response. Error bars in **(b, c, d, e, f, g, h, i)** represent Mean +/- SD. One-way ANOVA with Tukey's multiple comparisons test **(d, e, g, h, i)** and 2-tailed paired t-test **(b, c, f)** were used. Source data are provided as a Source Data file.

Supplementary Figure 6

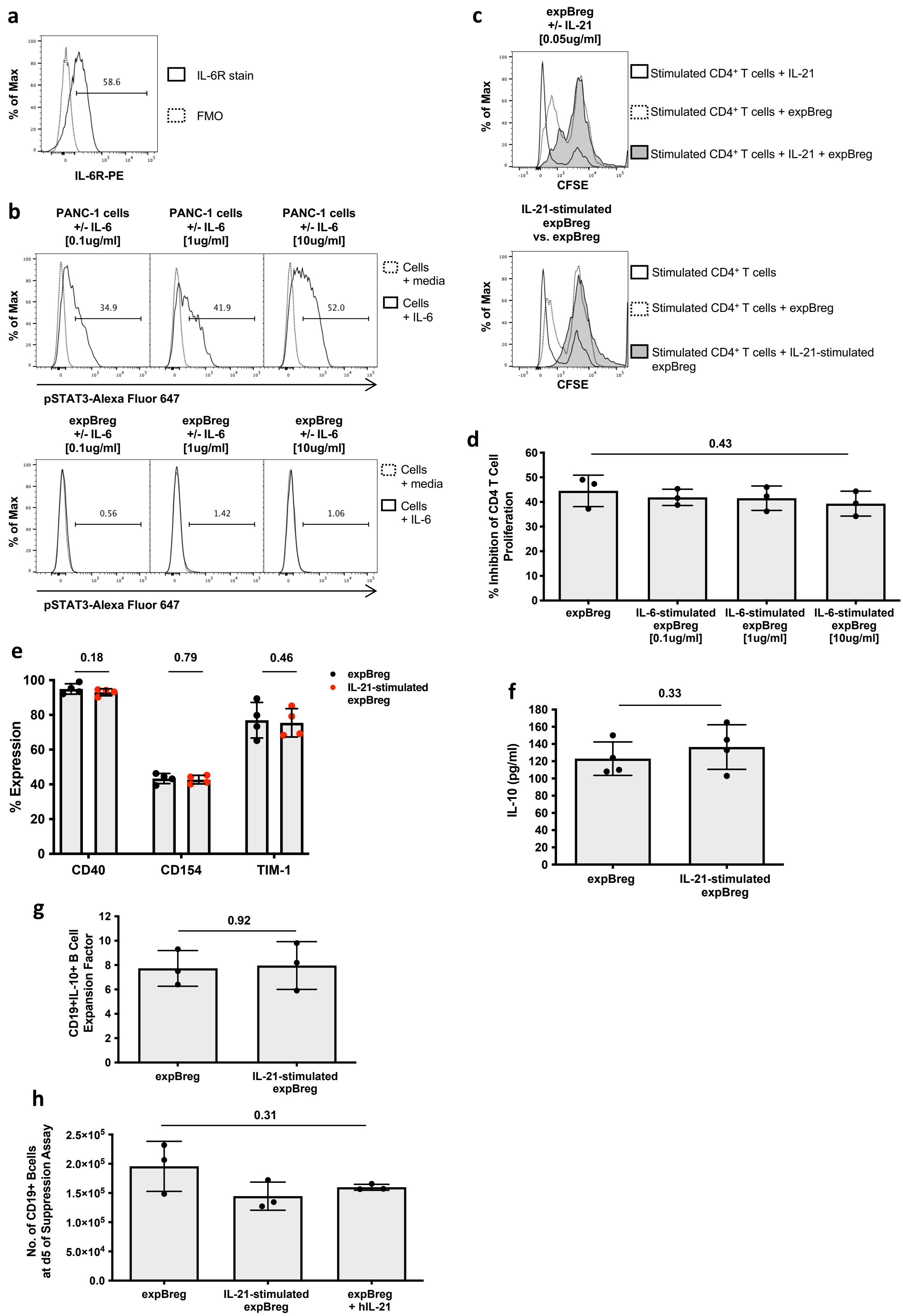

Supplementary Figure 6

(a) Representative histograms of live CD19^{+} expBreg are shown to demonstrate extracellular surface expression of IL-6 receptor (IL-6R). Gating is on fluorescence minus one controls (FMO). **(b)** Representative histograms of % STAT3 phosphorylation (pSTAT3) are presented when expBreg or an immortalised PANC-1 cell line were incubated with media or IL-6 ([0.01µg/ml], [1ug/ml] or [10ug/ml]) for 10 minutes and stained for pSTAT3. The PANC-1 cell line served as a positive control for IL-6-induced STAT3 phosphorylation. **(c)** Representative histograms of live CD4^{+}CFSE^{+} T cells are presented when autologous CD4^{+}CFSE^{+} T cells and anti-CD3/CD28 beads were co-cultured with expBreg +/- IL-21. expBreg were either pre-incubated with IL-21 [0.05µg/ml] for the last 48hrs of the 7-day expansion co-culture (IL-21-stimulated expBreg) prior to addition to the suppression assay at day-0, or IL-21 [0.05µg/ml] was directly added at day-0 of the suppression assay +/- expBreg (expBreg + hIL-21). **(d)** Summarised data of % inhibition of CD4^{+} T cell proliferation are presented when autologous CD4^{+}VPD^{+} T cells and anti-CD3/CD28 beads were co-cultured alone or with expBreg. expBreg had been pre-incubated with IL-6 ([0.01µg/ml], [1ug/ml] or [10ug/ml]) for the last 48hrs of the 7-day expansion co-culture (IL-6-stimulated expBreg). Data from experiments performed with cells from different healthy donors ($n = 3$) are presented. Each dot is an individual response. **(e)** Summarised data are presented which demonstrate % expression of CD40, CD154 and TIM-1 by expBreg and IL-21-stimulated expBreg. Data from experiments performed with cells from different healthy donors ($n = 4$) are presented. Each dot is an individual response. **(f)** Summarised data of IL-10 production by expBreg and IL-21-stimulated expBreg. Data from experiments performed with cells from different healthy donors ($n = 4$) are presented. Each dot is an individual response. **(g)** Summarised data of expansion factor of expBreg +/- IL-21 for the last 48hrs of the 7-day expansion co-culture. Data from experiments performed with cells from different healthy donors ($n = 3$) are presented. Each dot is an individual response. **(h)** Summarised data of number of CD19^{+} B cells at day-5 of suppression assay when expBreg, IL-21-stimulated expBreg or expBreg + IL-21 had been added at day-0 of suppression assay. Data from experiments performed with cells from different healthy donors ($n = 3$) are presented. Error bars in **(d, e, f, g, h)** represent Mean +/- SD. One-way ANOVA with Tukey's multiple comparisons test **(d, h)** and 2-tailed paired t-test **(e, f, g)** were used. Source data are provided as a Source Data file.

Supplementary Figure 7

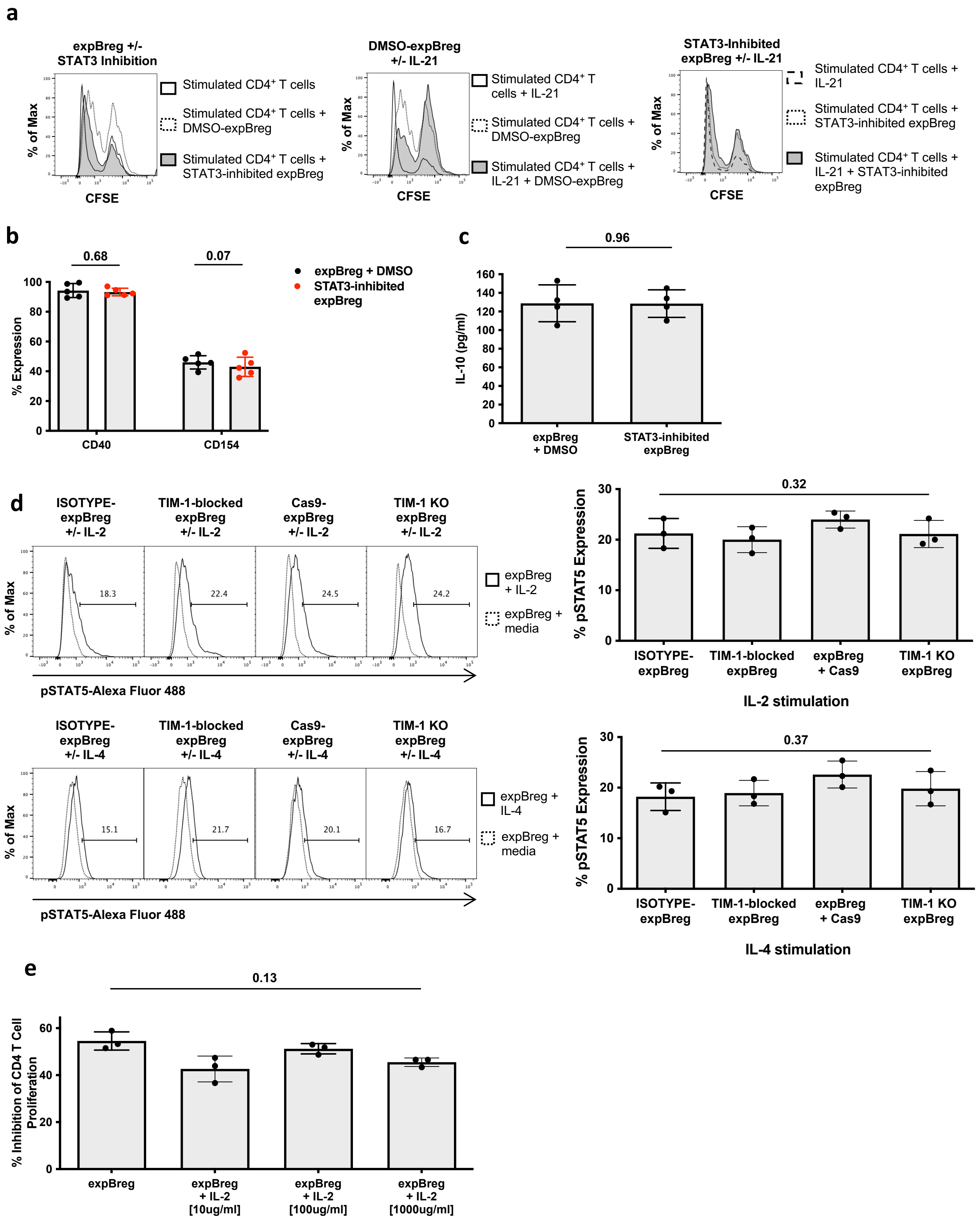

Supplementary Figure 7

(a) Representative histograms of live $CD4^{+}CFSE^{+}$ T cells are presented when autologous $CD4^{+}CFSE^{+}$ T cells and anti-CD3/CD28 beads were co-cultured with expBreg +/- STAT3 inhibitor +/- IL-21 [0.05µg/ml]. expBreg were incubated for 2 hours with the STAT3 inhibitor WP 1066 [10µM] +/- hIL-21 [0.05µg/ml], or DMSO +/- hIL-21 [0.05µg/ml], washed and suppressive function analysed. **(b)** Summarised data are presented which demonstrate expression of CD40 and CD154 in the presence of STAT3 inhibition or DMSO. Data from experiments performed with cells from different healthy donors ($n = 5$) are presented. Each dot is an individual response. **(c)** Summarised data of IL-10 production by expBreg are presented when in the presence of STAT3 inhibition or DMSO. Data from experiments performed with cells from different healthy donors ($n = 4$) are presented. Each dot is an individual response. **(d)** Representative histograms of $CD19^{+}$ B cells and summarised data of % STAT5 phosphorylation (pSTAT5) are presented when Isotype-expBreg, TIM-blocked-expBreg, Cas9-expBreg or TIM-1 KO expBreg were incubated with media, IL-2 [10^{4} U/ml] or IL-4 [1µg/ml] for 10 minutes and stained for pSTAT5. Data from experiments performed with cells from different healthy donors ($n = 3$) are presented. Each dot is an individual response. **(e)** Summarised data of % inhibition of $CD4^{+}$ T cell proliferation are presented when autologous $CD4^{+}CFSE^{+}$ T cells and anti-CD3/CD28 beads were co-cultured with expBreg +/- IL-2. IL-2 was added at day-0 of the suppression assay, at different concentrations ([10ug/ml], [100ug/ml], 1000ug/ml]). Data from experiments performed with cells from different healthy donors ($n = 3$) are presented. Each dot is an individual response. Error bars in **(b, c, d, e)** represent Mean +/- SD. One-way ANOVA with Tukey's multiple comparisons test **(d, e)** and 2-tailed paired t-test **(b, c)** were used. Source data are provided as a Source Data file.

Supplementary Figure 8

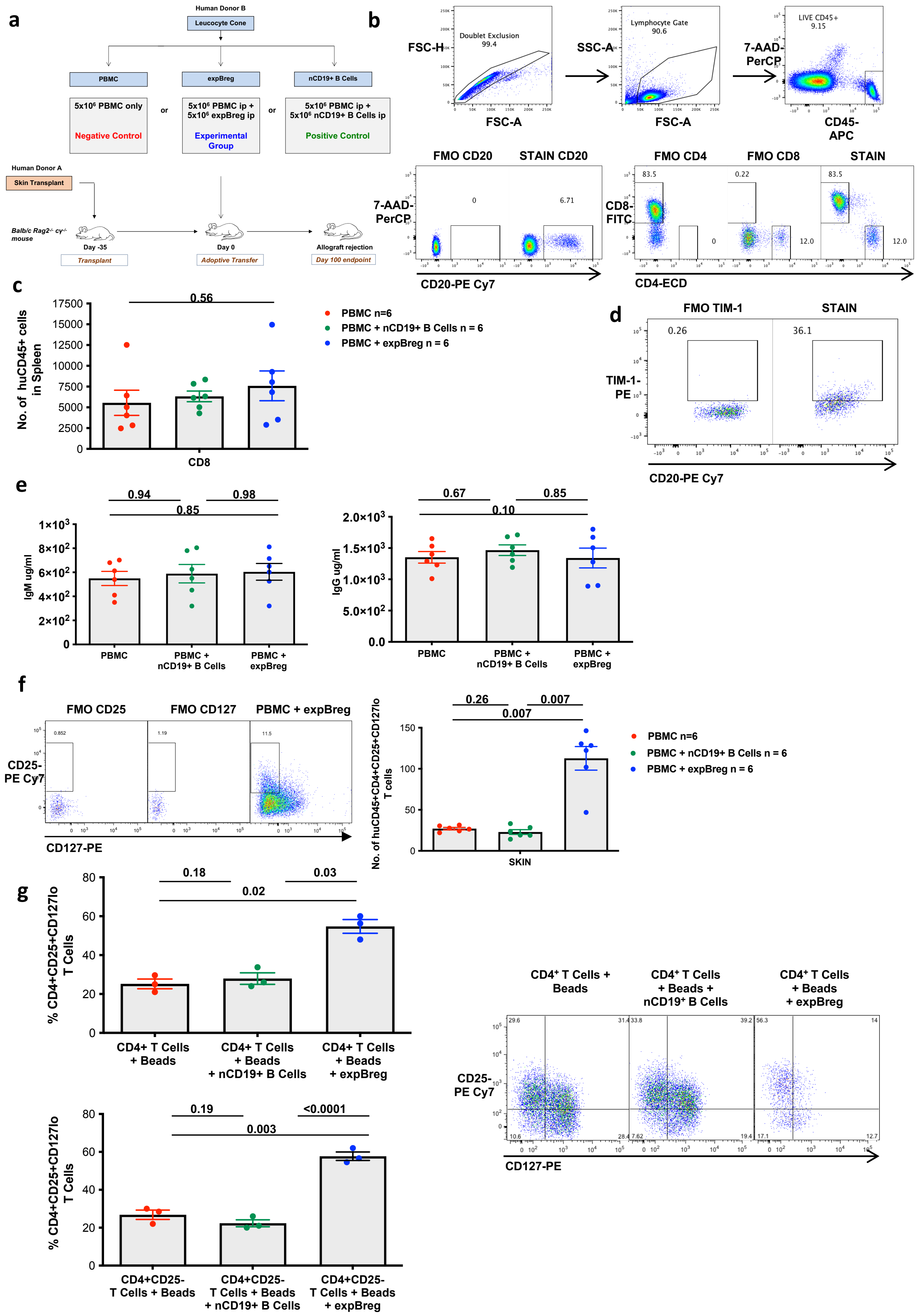

Supplementary Figure 8

(a) Humanised mouse model of human skin transplantation experimental set-up. **(b)** Gating strategy and representative FACS plots of live $huCD45^{+}$ cells in human skin allograft are presented. Gating is on fluorescence minus one controls (FMO). **(c)** Summarised data of the number of $huCD45^{+}CD8^{+}$ T cells in spleen of mice receiving PBMC +/- $nCD19^{+}$ B cells or expBreg are presented. n=6 per group per experiment, one of three independent experiments is shown. Each dot is an individual mouse response. Each experiment used a different HLA-mismatched human donor pair. **(d)** Representative FACS plots of live $huCD45^{+}CD20^{+}TIM\text{-}1^{+}$ B cells in human skin allograft are presented. Gating is on FMO controls. **(e)** Summarised data of serum levels of human IgM and IgG in peripheral blood of mice receiving PBMC +/- $nCD19^{+}$ B cells or expBreg are presented. n=6 per group per experiment, one of three independent experiments is shown. Each dot is an individual mouse response. Each experiment used a different HLA-mismatched human donor pair. **(f)** Representative FACS plots and summarised data of the number of live $huCD45^{+}CD4^{+}CD25^{+}CD127^{lo}$ putative Treg in human skin allograft of mice receiving PBMC +/- $nCD19^{+}$ B cells or expBreg are presented. n=6 per group per experiment, one of three independent experiments is shown. Each dot is an individual mouse response. Each experiment used a different HLA-mismatched human donor pair. **(g)** Representative FACS plots and summarised data of live $CD4^{+}$ T cells demonstrating % of putative Treg when expBreg and anti CD3/CD28 beads are co-cultured with CD4+ T cells or $CD4^{+}CD25^{-}$ T cells *in vitro* for 5 days, when compared to controls. Data from experiments performed with cells from different healthy donors ($n = 3$) are presented. Each dot is an individual response. Error bars in **(c, e, f)** represent Mean +/- SEM. Error bars in **(g)** represent Mean +/- SD. One-way ANOVA with Tukey's multiple comparisons test **(c, e, f, g)** were used. Source data are provided as a Source Data file.

Supplementary Figure 9

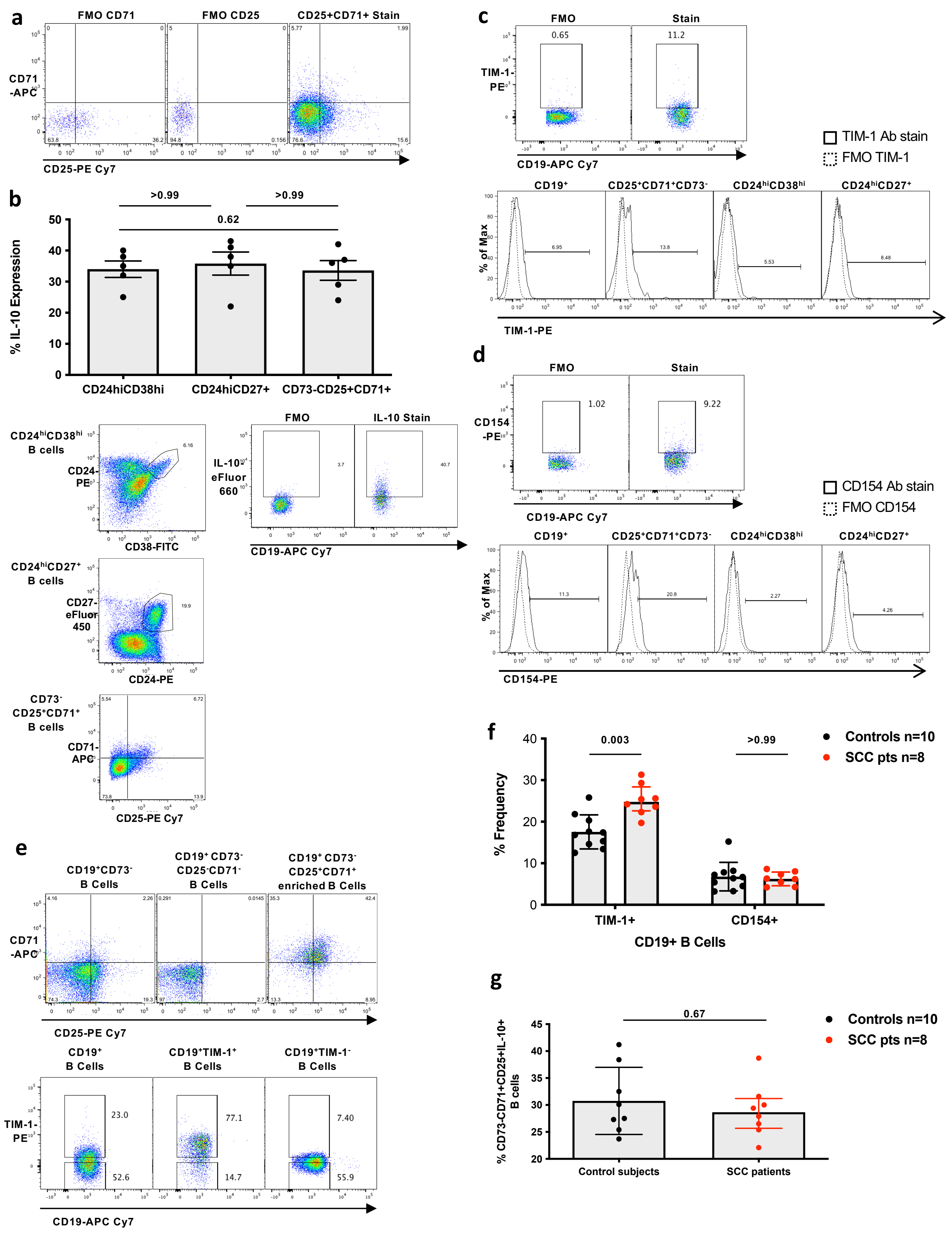

Supplementary Figure 9

(a) Representative FACS plots of live $CD19^{+}CD73^{-}$ B cells to demonstrate gating strategy for $CD19^{+}CD73^{-}CD25^{+}CD71^{+}$ B Cells from human peripheral blood. Gating is on fluorescence minus one controls (FMO). **(b)** Representative FACS plots and summarised data of % IL-10 expression by $CD24^{hi}CD38^{hi}$, $CD24^{hi}CD27^{+}$ and $CD73^{-}CD25^{+}CD71^{+}$ Breg subsets from human peripheral blood. Gating is on FMO controls. Data from experiments performed with cells from different healthy donors ($n = 5$) are presented. Each dot is an individual response. **(c)** Representative FACS plots of % TIM-1^{+} B cells in whole $CD19^{+}$ B cells, $CD73^{-}CD25^{+}CD71^{+}$, $CD24^{hi}CD38^{hi}$ and $CD24^{hi}CD27^{+}$ B cell subsets in peripheral blood of healthy human donors. Gating is on FMO controls. **(d)** Representative FACS plots of % $CD154^{+}$ B cells in whole $CD19^{+}$ B cells, $CD73^{-}CD25^{+}CD71^{+}$, $CD24^{hi}CD38^{hi}$ and $CD24^{hi}CD27^{+}$ B cell subsets in peripheral blood of healthy human donors. Gating is on FMO controls. **(e)** Representative FACS plots of FACS-sorted $CD73^{-}CD25^{-}CD71^{-}$, $CD73^{-}CD25^{+}CD71^{+}$ enriched, TIM-1^{+} and TIM-1^{-} B cells from human peripheral blood. **(f)** Summarised data of % TIM-1^{+} and $CD154^{+}$ B cells within $CD19^{+}$ B cells in peripheral blood of healthy human donors (n=10) and age-matched patients with SCC (n=8). Each dot is an individual response. **(g)** Summarised data of % IL-10 expression within $CD73^{-}CD25^{+}CD71^{+}$ B cells in peripheral blood of healthy human donors (n=10) and age-matched patients with SCC (n=8). Each dot is an individual response**.** Error bars in **(b, f, g)** represent Median + interquartile range. One way ANOVA with Dunn's multiple comparisons test **(b)** and 2-tailed Mann Whitney test **(f, g)** were used. Source data are provided as a Source Data file.

Supplementary Figure 10

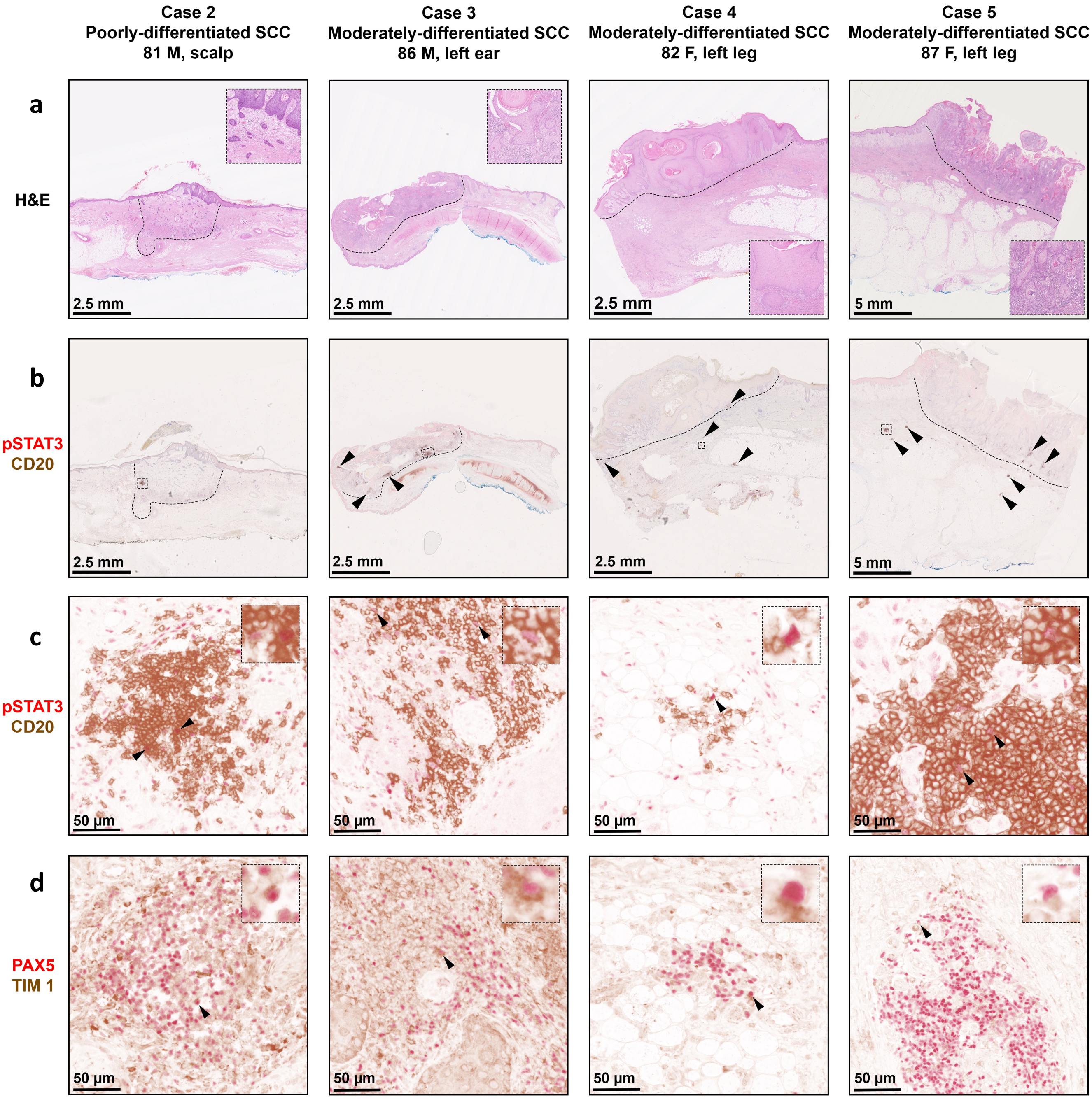

Supplementary Figure 10

(a) Hematoxylin and eosin (H&E) stained histological sections demonstrating invasive squamous cell carcinomas in an additional four patients (hashed line delineates tumour boundary; higher magnification image demonstrating representative histological features in top or bottom right-hand corners). **(b)** Double immunohistochemistry staining for pSTAT3 (red) and CD20 (brown) demonstrates the peritumoral accumulation of $CD20^+$ B cells (arrows), predominantly arranged as clusters, both within the tumours themselves and in proximity to their invasive margins (hashed line). **(c)** Higher magnification images of the annotated B cell clusters shown in (b) (hashed boxes), demonstrating double positive cells (arrows). **(d)** On the adjacent section, a single cell double stained for PAX5 (a master regulator of B cell development; red) and TIM 1 (brown) is identified in each cluster (arrows; higher magnification images of these cells in top right-hand corners). Each case in **(a, b, c, d)** represents 1 patient (n=4 patients).

Supplementary Table 1

Antibody (Human)	Fluorochrome	Concentration	Supplier	Catalogue Number
**CD19**	APC-Cy7	1:100	BD Pharmingen	557791
**CD25**	PE-Cy7	1:100	BD Pharmingen	557741
**CD24**	PE	1:100	BD Pharmingen	555428
**CD38**	FITC	1:100	BD Pharmingen	555459
**CD127**	PE	1:100	BD Pharmingen	557938
**IgD**	PE-Cy7	1:100	BD Pharmingen	561314
**pSTAT3**	Alexa Fluor 647	1:6	BD Pharmingen	557815
**pSTAT5**	Alexa Fluor 488	1:6	BD Pharmingen	562077
**CD20**	PE-Cy7	1:100	eBioscience	25-0209-42
**CD4**	PE-Texas Red	1:100	eBioscience	61-0049-42
**CD8**	FITC	1:100	eBioscience	11-0088-42
**γCR**	PE	1:100	eBioscience	12-1329-41
**CD27**	eFluor 450	1:100	eBioscience	48-0279-42
**CD138**	eFluor 450	1:100	eBioscience	17-1389-41
**CD1d**	APC	1:100	eBioscience	17-0016-42
**CD5**	FITC	1:100	eBioscience	11-0058-42
**CD21**	eFluor 450	1:100	eBioscience	9048-0219-025
**CD71**	APC	1:100	eBioscience	17-0719-41
**CD73**	eFluor 450	1:100	eBioscience	48-0739-41
**LAP**	PE	1:100	eBioscience	12-9829-41
**IL-10**	eFluor 660	1:4	eBioscience	50-7108-41
**TNFα**	FITC	1:4	eBioscience	11-7349-41
**IFNγ**	PE	1:4	eBioscience	12-7319-41
**CD154**	PE	1:100	eBioscience	12-1548-42
**IgM**	PE	1:100	eBioscience	12-9998-42
**CD40**	APC	1:100	eBioscience	17-0409-41
**CD80**	FITC	1:100	eBioscience	11-0809-42
**CD86**	PE	1:100	eBioscience	12-0869-42
**FAS-L**	PE	1:100	eBioscience	12-9919-41
**PD-L1**	PE-Cy7	1:100	eBioscience	25-5983-41
**PD-L2**	APC	1:100	eBioscience	17-5888-41
**TIM-1**	PE	1:100	Biolegend	353903
**IL-10R**	APC	1:100	Biolegend	308811
**CD122**	APC	1:100	Biolegend	339007
**IL-6Rα**	PE	1:100	Biolegend	352803
**CD154**	FITC	1:100	Biolegend	310804
**CD10**	PE-Texas Red	1:100	Beckman Coulter	41116015
**CD45**	APC	1:100	Invitrogen	MHCD45054
**CD154**	APC	1:100	R&D Systems	FAB617A
**CD25**	PE	1:100	R&D Systems	FAB1020P-025
**CD154**	Unconjugated	[10μg/ml]	R&D Systems	MAB617
**CD40**	Unconjugated	[10μg/ml]	R&D Systems	MAB6322
**IL-10**	Unconjugated	[10μg/ml]	R&D Systems	MAB217
**IL-10Rα**	Unconjugated	[10μg/ml]	R&D Systems	MAB274
**CD122**	Unconjugated	[10μg/ml]	R&D Systems	MAB224
**CD25**	Unconjugated	[10μg/ml]	R&D Systems	MAB223

Antibody (Human)	Fluorochrome	Concentration	Supplier	Catalogue Number
**CD80**	Unconjugated	[10µg/ml]	R&D Systems	MAB140
**CD86**	Unconjugated	[10µg/ml]	R&D Systems	MAB141
**FASL**	Unconjugated	[10µg/ml]	R&D Systems	MAB126
**PD-1**	Unconjugated	[10µg/ml]	R&D Systems	AF1086
**IL-6**	Unconjugated	[10µg/ml]	R&D Systems	MAB2061
**IL-6R**	Unconjugated	[10µg/ml]	R&D Systems	MAB227R
**$IgG_{1\kappa}$**	Unconjugated	[10µg/ml]	R&D Systems	MAB002
**$IgG_{2a\kappa}$**	Unconjugated	[10µg/ml]	R&D Systems	MAB004
**CD25**	Unconjugated	[10µg/ml]	R&D Systems	MAB1020
**TIM-1**	Unconjugated	[10µg/ml]	Biolegend	353902
**CD20**	Unconjugated	1:100	Leica Biosystems	PA0200
**pSTAT3**	Unconjugated	1:100	Abcam	ab76315
**TIM-1**	Unconjugated	1:100	Abcam	ab47635
**PAX5**	Unconjugated	1:500	Abcam	ab109443
