# Supplementary material for: Ex vivo-expanded human CD19+TIM-1+ regulatory B cells suppress immune responses in vivo and are dependent upon the TIM-1/STAT3 axis
Source: Nat Commun. 2022 Jun 3;13:3121. doi: 10.1038/s41467-022-30613-z (PMC9166804; doi:10.1038/s41467-022-30613-z)
Supplement: Supplementary file 2 — Reporting Summary [file 41467_2022_30613_MOESM2_ESM.pdf]

nature portfolio

Corresponding author(s): Sushma Shankar

Last updated by author(s): Apr 1, 2022

Reporting Summary

Nature Portfolio wishes to improve the reproducibility of the work that we publish. This form provides structure for consistency and transparency in reporting. For further information on Nature Portfolio policies, see our Editorial Policies and the Editorial Policy Checklist.

Statistics

For all statistical analyses, confirm that the following items are present in the figure legend, table legend, main text, or Methods section.

n/a	Confirmed	
☐	☒	The exact sample size (*n*) for each experimental group/condition, given as a discrete number and unit of measurement
☐	☒	A statement on whether measurements were taken from distinct samples or whether the same sample was measured repeatedly
☐	☒	The statistical test(s) used AND whether they are one- or two-sided<br>*Only common tests should be described solely by name; describe more complex techniques in the Methods section.*
☐	☒	A description of all covariates tested
☐	☒	A description of any assumptions or corrections, such as tests of normality and adjustment for multiple comparisons
☐	☒	A full description of the statistical parameters including central tendency (e.g. means) or other basic estimates (e.g. regression coefficient) AND variation (e.g. standard deviation) or associated estimates of uncertainty (e.g. confidence intervals)
☐	☒	For null hypothesis testing, the test statistic (e.g. F, t, r) with confidence intervals, effect sizes, degrees of freedom and P value noted<br>*Give P values as exact values whenever suitable.*
☒	☐	For Bayesian analysis, information on the choice of priors and Markov chain Monte Carlo settings
☒	☐	For hierarchical and complex designs, identification of the appropriate level for tests and full reporting of outcomes
☒	☐	Estimates of effect sizes (e.g. Cohen's *d*, Pearson's *r*), indicating how they were calculated

Our web collection on statistics for biologists contains articles on many of the points above.

Software and code

Policy information about availability of computer code

Data collection	Flow cytometric data were acquired using BD Canto II (BD Biosciences, UK), and analysed using FlowJo software v10.7.1 (Flowjo Enterprise, USA). This is stated under 'Flow Cytometry,' in the Methods section of the manuscript.
Data analysis	Statistical analysis was performed using Graphpad Prism v9.0 software. This is stated under 'Statistical Analysis,' in the Methods section of the manuscript.

For manuscripts utilizing custom algorithms or software that are central to the research but not yet described in published literature, software must be made available to editors and reviewers. We strongly encourage code deposition in a community repository (e.g. GitHub). See the Nature Portfolio guidelines for submitting code & software for further information.

Data

Policy information about availability of data

All manuscripts must include a data availability statement. This statement should provide the following information, where applicable:
- Accession codes, unique identifiers, or web links for publicly available datasets
- A description of any restrictions on data availability
- For clinical datasets or third party data, please ensure that the statement adheres to our policy

'The authors declare that all relevant data supporting the findings of this study are available within the paper and its supplementary information files. Source data are provided with this paper. The raw numbers for charts and graphs are available in the Source Data file whenever possible.'
(The above statement is found under 'Data Availability,' in the Methods section of the manuscript)

Field-specific reporting

Please select the one below that is the best fit for your research. If you are not sure, read the appropriate sections before making your selection.

[x] Life sciences [ ] Behavioural & social sciences [ ] Ecological, evolutionary & environmental sciences

For a reference copy of the document with all sections, see nature.com/documents/nr-reporting-summary-flat.pdf

Life sciences study design

All studies must disclose on these points even when the disclosure is negative.

Sample size	Mouse numbers and numbers of human donors for the in vitro experiments are calculated based on the minimum required to achieve statistical significance (for in vivo work, minimum of n=5/group for graft survival analysis and n=3 for mechanistic analysis). These sizes are calculated based on the magnitude of responses recorded when manipulating the immune system in similar experiments from our previously published work and elsewhere in the literature:<br><br>Issa, F., et al., Ex vivo-expanded human regulatory T cells prevent the rejection of skin allografts in a humanized mouse model. Transplantation, 2010. 90(12): p. 1321-7.<br>Nadig, S.N., et al., In vivo prevention of transplant arteriosclerosis by ex vivo-expanded human regulatory T cells. Nat Med, 2010. 16(7): p. 809-13.<br>Festing, M.F., The design and statistical analysis of animal experiments. ILAR J, 2002. 43(4): p. 191-3
Data exclusions	No data was excluded from the analyses.<br>Human patients were recruited to the study based on an exclusion criteria that is defined under 'Procurement of Human Skin,' in the Methods section: 'Exclusion criteria included donors who were taking immunosuppressive medication, donors with a primary inflammatory condition or donors with any current or past history of malignancy.' Exclusion criteria for human patients is also defined in 'Human Donors,' in the Methods sections: 'participants who had been diagnosed with an autoimmune or inflammatory condition, or a malignancy, were excluded.'
Replication	All in vitro experimental conditions were set up in triplicate as a minimum number of technical repeats. Each in vitro experiment with human samples was conducted between 3-5 times, each with a different human donor. In vivo experiments involving humanised mice were set up with 6 mice in each experimental condition per experiment. Each mouse experiment was conducted a minimum of 3 times, each time using a different HLA-mismatched human donor pair. This is outlined in the figure legends.
Randomization	In mouse experiments, animals were randomly allocated to each experimental group. All mice were of the same species, sex and age. Mice of different experimental groups were co-housed. This is stated in the 'Mice' section of the Methods section.
Blinding	For assessment of human skin allografts in mouse experiments, blind assessment of the grafts was undertaken. This is stated under 'Skin grafting,' in the Methods section: 'graft assessment was performed independently by two researchers who were both blinded to experimental group allocations.'

Reporting for specific materials, systems and methods

We require information from authors about some types of materials, experimental systems and methods used in many studies. Here, indicate whether each material, system or method listed is relevant to your study. If you are not sure if a list item applies to your research, read the appropriate section before selecting a response.

Materials & experimental systems

n/a	Involved in the study	
[ ]	[x]	Antibodies
[ ]	[x]	Eukaryotic cell lines
[x]	[ ]	Palaeontology and archaeology
[ ]	[x]	Animals and other organisms
[ ]	[x]	Human research participants
[x]	[ ]	Clinical data
[x]	[ ]	Dual use research of concern

Methods

n/a	Involved in the study	
[x]	[ ]	ChIP-seq
[ ]	[x]	Flow cytometry
[x]	[ ]	MRI-based neuroimaging

Antibodies

Antibodies used	All antibodies used are characterised in Supplementary Table 1, in the Supplementary information (this is referred to within the Methods section):<br><br>Antibody (Human); Fluorochrome; Concentration; Supplier Catalogue Number;<br>CD19 APC-Cy7 1:100 BD Pharmingen 557791

CD25 PE-Cy7 1:100 BD Pharmingen 557741
CD24 PE 1:100 BD Pharmingen 555428
CD38 FITC 1:100 BD Pharmingen 555459
CD127 PE 1:100 BD Pharmingen 557938
IgD PE-Cy7 1:100 BD Pharmingen 561314
pSTAT3 Alexa Fluor 647 1:6 BD Pharmingen 557815
pSTAT5 Alexa Fluor 488 1:6 BD Pharmingen 562077
CD20 PE-Cy7 1:100 eBioscience 25-0209-42
CD4 PE-Texas Red 1:100 eBioscience 61-0049-42
CD8 FITC 1:100 eBioscience 11-0088-42
γCR PE 1:100 eBioscience 12-1329-41
CD27 eFluor 450 1:100 eBioscience 48-0279-42
CD138 eFluor 450 1:100 eBioscience 17-1389-41
CD1d APC 1:100 eBioscience 17-0016-42
CD5 FITC 1:100 eBioscience 11-0058-42
CD21 eFluor 450 1:100 eBioscience 9048-0219-025
CD71 APC 1:100 eBioscience 17-0719-41
CD73 eFluor 450 1:100 eBioscience 48-0739-41
LAP PE 1:100 eBioscience 12-9829-41
IL-10 eFluor 660 1:4 eBioscience 50-7108-41
TNFα FITC 1:4 eBioscience 11-7349-41
IFNγ PE 1:4 eBioscience 12-7319-41
CD154 PE 1:100 eBioscience 12-1548-42
IgM PE 1:100 eBioscience 12-9998-42
CD40 APC 1:100 eBioscience 17-0409-41
CD80 FITC 1:100 eBioscience 11-0809-42
CD86 PE 1:100 eBioscience 12-0869-42
FAS-L PE 1:100 eBioscience 12-9919-41
PD-L1 PE-Cy7 1:100 eBioscience 25-5983-41
PD-L2 APC 1:100 eBioscience 17-5888-41
TIM-1 PE 1:100 Biolegend 353903
IL-10R APC 1:100 Biolegend 308811
CD122 APC 1:100 Biolegend 339007
IL-6Rα PE 1:100 Biolegend 352803
CD154 FITC 1:100 Biolegend 310804
CD10 PE-Texas Red 1:100 Beckman Coulter 41116015
CD45 APC 1:100 Invitrogen MHCD45054
CD154 APC 1:100 R&D Systems FAB617A
CD25 PE 1:100 R&D Systems FAB1020P-025
CD154 Unconjugated [10µg/ml] R&D Systems MAB617
CD40 Unconjugated [10µg/ml] R&D Systems MAB6322
IL-10 Unconjugated [10µg/ml] R&D Systems MAB217
IL-10Rα Unconjugated [10µg/ml] R&D Systems MAB274
CD122 Unconjugated [10µg/ml] R&D Systems MAB224
CD25 Unconjugated [10µg/ml] R&D Systems MAB223
CD80 Unconjugated [10µg/ml] R&D Systems MAB140
CD86 Unconjugated [10µg/ml] R&D Systems MAB141
FASL Unconjugated [10µg/ml] R&D Systems MAB126
PD-1 Unconjugated [10µg/ml] R&D Systems AF1086
IL-6 Unconjugated [10µg/ml] R&D Systems MAB2061
IL-6R Unconjugated [10µg/ml] R&D Systems MAB227R
IgG1κ Unconjugated [10µg/ml] R&D Systems MAB002
IgG2aκ Unconjugated [10µg/ml] R&D Systems MAB004
CD25 Unconjugated [10µg/ml] R&D Systems MAB1020
TIM-1 Unconjugated [10µg/ml] Biolegend 353902
CD20 Unconjugated 1:100 Leica Biosystems PA0200
pSTAT3 Unconjugated 1:100 Abcam ab76315
TIM-1 Unconjugated 1:100 Abcam ab47635
PAX5 Unconjugated 1:500 Abcam ab109443

Validation

Each antibody used has been validated for species and application by the manufacturer, as stated on their websites. For all flow cytometry analysis, staining using these antibodies has been confirmed with fluorescence minus one controls and/or isotype controls. Representative FACS plots for all staining is provided in the Main and Supplementary Figures.

Eukaryotic cell lines

Policy information about cell lines

Cell line source(s)	CD154+ Chinese Hamster Ovary (CHO) Cell line: Gift of Professor Claudia Mauri, University College London. PANC-1 cell line: Purchased from ATCC. This information is stated under 'Culture of Chinese Hamster Ovary (CHO) cells' and 'PANC-1 cells' sections in the Methods section.
Authentication	Cell lines were not authenticated. Human CD154 expression by CD154+ CHO cell line was confirmed by flow cytometry
Mycoplasma contamination	All cell lines tested negative for mycoplasma. This information is stated under 'Culture of Chinese Hamster Ovary (CHO) cells' and 'PANC-1 cells' sections in the Methods section.
Commonly misidentified lines (See ICLAC register)	Neither cell line used in this manuscript are on the ICLAC register as commonly misidentified cell lines.

Animals and other organisms

Policy information about studies involving animals; ARRIVE guidelines recommended for reporting animal research

Laboratory animals	Balb/c Rag2-/- cγ-/- mice were obtained from Charles River Laboratories, stock number 14593, and were housed under specific pathogen-free conditions.. Mice were housed in individually ventilated cages and handled with gloves. Experimental and control mice were co-housed. Female mice were used for all experiments. At the time of the first experimental procedure, mice were between the ages of 6 and 12 weeks. This information is under the 'Mice' section in the Methods.
Wild animals	The study did not involve wild animals.
Field-collected samples	The study did not involve samples collected from the field.
Ethics oversight	All experiments were performed using protocols approved by the Committee on Animal Care and Ethical Review at the University of Oxford and in accordance with the UK Animals (Scientific Procedures) Act 1986, under project license PPL P88695354 and personal license PIL 30/9202, permission granted by the UK Home Office. This information is stated under the 'Mice' section in the Methods.<br>Human tissue for use in animal experiments was obtained with informed patient consent and ethical approval from the Oxfordshire Research Ethics Committee (REC B), study number 07/H0605/130. This information is stated under 'Procurement of human skin,' section in the Methods.

Note that full information on the approval of the study protocol must also be provided in the manuscript.

Human research participants

Policy information about studies involving human research participants

Population characteristics	Blood samples from patients with cutaneous squamous cell carcinoma (SCC) were taken at time-points ranging from 0 to 755 days after the SCC diagnosis had been made (mean time-point of sample was 392 days). SCC patients were age-matched to healthy controls (SCC patients: mean age 81 yrs, age range 72-90 yrs. Healthy controls: mean age 77 yrs, age range 68-86 yrs). All SCC patients and healthy controls were male. When recruiting healthy controls for comparison to SCC patients, participants who had been diagnosed with an autoimmune or inflammatory condition, or a malignancy, were excluded. This information is stated in 'Human donors,' section in the methods.<br>Characteristics of human donors of skin for transplantation into humanised mice, are described under 'Procurement of human skin,' section in the Methods: 'Exclusion criteria included donors who were taking immunosuppressive medication, donors with a primary inflammatory condition or donors with any current or past history of malignancy. Donor age ranged from 28 to 53 years (median 43 years) and donors were all male'<br>Human blood samples for all other analyses were obtained from NHS Blood & Transplant as anonymous samples from healthy donors. We do not have access to demographic data for these donors. This is stated in 'Human donors,' section in the methods.
Recruitment	Participants with cutaneous squamous cell carcinoma (SCC) and age- and sex-matched controls without SCC were recruited from the Dermatology department within Oxford University Hospitals NHS Foundation Trust, by the clinical and research teams. Human blood samples from healthy donors were provided by NHS Blood & Transplant.
Ethics oversight	Blood samples from patients with cutaneous squamous cell carcinoma (SCC) and from healthy human donors, were obtained with informed consent and ethical approval from the NHS Research Ethics Committee, study numbers 12/WS/0288 and 14/SC/0091.

Note that full information on the approval of the study protocol must also be provided in the manuscript.

Flow Cytometry

Plots

Confirm that:

- [x] The axis labels state the marker and fluorochrome used (e.g. CD4-FITC).
- [x] The axis scales are clearly visible. Include numbers along axes only for bottom left plot of group (a 'group' is an analysis of identical markers).
- [x] All plots are contour plots with outliers or pseudocolor plots.
- [x] A numerical value for number of cells or percentage (with statistics) is provided.

Methodology

Sample preparation	Cell-surface marker staining: Cells were first incubated with human sera for 15 minutes at 4°C to reduce non-specific antibody binding. Cells were then incubated with the appropriate fluorochrome-coupled mAbs for 45 minutes at 4°C in the dark. Cells were then washed with PBS and spun for 5 minutes at 1500rpm at 4°C before flow cytometry analysis. Fluorescence-minus-one (FMO) controls were used as standard controls to permit accurate gating of positive and negative populations.<br><br>Intracellular staining: After appropriate cell-surface staining, cells were permeabilised using a BD Cytofix/Cytoperm Kit (BD Biosciences, UK) in accordance with manufacturer's guidelines. Cells were then incubated with either the fluorochrome-coupled mAb under interrogation or the appropriate isotype control for 1 hour at 4°C in the dark. In some experiments, fluorescence-minus-one controls (FMO) were used. Cells were washed with PBS and spun for 5 minutes at 1500rpm at 4°C before flow cytometry analysis. In order to measure expression of IL-10, cells were first incubated for 6 hours with PMA, ionomycin and monensin (PIM) in c.RPMI before staining.<br><br>pSTAT3 and pSTAT5 staining: expBreg cells were harvested from expansion co-cultures, initially rested in c.RPMI for 90 minutes and then washed to allow any existing pSTAT3 or pSTAT5 to degrade. 0.1 x106 expBreg cells were subsequently incubated in 100µl c.RPMI for 10 minutes at 37°C, with exogenous hIL-10 [0.01µg/ml], hIL-21 [0.05µg/ml], hIL-2 [104 U/ml] or hIL-4 [1µg/ml] (or c.RPMI as a control) in 96-well, v-bottom plates. 100µl/well of Cytofix fixation Buffer (BD Biosciences, UK) was added to cell culture for an additional 10-minute incubation period at 37°C. Cells were subsequently spun for 5 minutes at 1500rpm at 4°C and supernatants decanted. 100µl/well of Phosflow Perm Buffer (BD Biosciences, UK) was added to cell culture and cells were incubated for 30 minutes on ice. Cells were then washed twice with PBS at 1500 rpm for 5 minutes at 4°C before staining with Alexa Fluor 647-conjugated pSTAT3 antibody (BD Pharmingen) or Alexa Fluor 488-conjugated pSTAT5 (pY694) antibody (BD Biosciences) for 1 hour at 4°C. Cells were washed with PBS at 1500 rpm for 5 minutes at 4°C before immediate FACS analysis. FMO controls were used to assist accurate gating. In some experiments, expBreg underwent IL-10 exposure and pSTAT3 staining without an initial 90-minute rest period. CD4+ T cells, nCD19+ B cells and unstained expBreg cells were included as relevant controls.
Instrument	Samples were acquired and sorted using a FACSAria IIu (BD Biosciences, UK) whilst flow cytometric data were acquired using BD Canto II (BD Biosciences, UK),
Software	Samples were analysed using FlowJo software v10.7.1 (Flowjo Enterprise, USA).
Cell population abundance	A minimum of 1 million cells were acquired per cell population fraction post-sorting. Purity was determined by flow cytometry following the sort. Purities of all post-sort fractions are stated within the main text of the manuscript<br>TIM-1+ expBreg 70.6% purity (Supplementary Figure 4b)<br>TIM-1- expBreg 71.7% purity (Supplementary Figure 4b)<br>CD25+ expBreg 91.3% purity (Supplementary Figure 4d)<br>CD25- expBreg 93.7% purity (Supplementary Figure 4d)<br>CD4+CD25+CD127lo Treg >94% purity (Main manuscript)
Gating strategy	For all FACS-acquired data, doublet exclusion was first performed by FSC-H/FSC-A.<br>For data in Main Figures 1, 2, 3, 4, 5, and Supplementary Figures 1, 2, 3, 4, 5, 6, 7, 9, all cells within the starting cell population (human lymphocytes) were included in the FSC/SSC gate. For data in Main Figures 7, a lymphocyte gate was first identified within the peripheral blood mononuclear cells (PBMC) starting population, within the FSC/SSC gate.<br>Dead cell exclusion was then performed based on 7AAD viability dye. Different human cell subsets were then identified based on cell surface markers.<br>For data acquired in mice experiments in Main Figure 6 and Supplementary Figure 8, the gating strategy is demonstrated in Supplementary Figure 8b. All mouse and human cells within the starting cell population were included in the FSC/SSC gate. Dead cell exclusion was then performed based on 7AAD viability dye. Human cells were identified by positive expression of human CD45. Different human cell subsets were then identified based on cell surface markers.<br>Fluorescence-minus-one (FMO) controls and Isotype controls were used as standard controls to permit accurate gating of positive and negative populations. This is specified for each analysis in the relevant Main and Supplementary Figure legends.

- [x] Tick this box to confirm that a figure exemplifying the gating strategy is provided in the Supplementary Information.
